# The PROSECCO server for chemical shift predictions in ordered and disordered proteins

**DOI:** 10.1007/s10858-017-0145-2

**Published:** 2017-11-08

**Authors:** Máximo Sanz-Hernández, Alfonso De Simone

**Affiliations:** Department of Life Sciences, Imperial College London, London, SW7 2AZ UK

**Keywords:** Biomolecular NMR, Chemical shift predictions, Disordered proteins

## Abstract

The chemical shifts measured in solution-state and solid-state nuclear magnetic resonance (NMR) are powerful probes of the structure and dynamics of protein molecules. The exploitation of chemical shifts requires methods to correlate these data with the protein structures and sequences. We present here an approach to calculate accurate chemical shifts in both ordered and disordered proteins using exclusively the information contained in their sequences. Our sequence-based approach, protein sequences and chemical shift correlations (PROSECCO), achieves the accuracy of the most advanced structure-based methods in the characterization of chemical shifts of folded proteins and improves the state of the art in the study of disordered proteins. Our analyses revealed fundamental insights on the structural information carried by NMR chemical shifts of structured and unstructured protein states.

**Electronic supplementary material:**

The online version of this article (doi:10.1007/s10858-017-0145-2) contains supplementary material, which is available to authorized users.

## Introduction

Biomolecular nuclear magnetic resonance (NMR) spectroscopy has emerged as a powerful technique to accurately characterize the structure and dynamics of proteins and other biomacromolecules (Kay 2005). In the context of intrinsically disordered proteins (IDPs), NMR has a unique ability to quantify transient interactions and conformational preferences of such elusive protein states both in vitro (Jensen et al. 2010; Stollar et al. 2012) and in the cellular environment (Felli et al. 2014; Waudby et al. 2013). The quantitative interpretation of NMR spectra toward the characterization of the structural properties and atomic fluctuations of protein molecules has attracted considerable interest in the biochemical community. In this context, significant progress has been achieved by using statistical mechanics to analyze NMR databases, which enabled the definition of new approaches to study protein structure (Bouvignies et al. 2011; Cavalli et al. 2007; Hafsa et al. 2015; Shen et al. 2008) and dynamics (Berjanskii and Wishart 2005, 2013; Boulton et al. 2014; Kim et al. 2017; Krieger et al. 2014; Masterson et al. 2010; Neudecker et al. 2012; Robustelli et al. 2012; Selvaratnam et al. 2011) such as those employing exclusively chemical shifts (CS) from solution (Clore and Schwieters 2003; Kuszewski et al. 2004; Sgourakis et al. 2011) and solid state NMR (Mollica et al. 2012; Robustelli et al. 2008). Indeed CS are extremely precise probes of the secondary structures in folded and disordered proteins and have been largely used to map backbone dihedral angles (Neal et al. 2006; Shen and Bax 2015a).

The interpretation of CS of folded proteins is based on the ability to correlate these experimental data with protein three-dimensional structure (Han et al. 2011; Kohlhoff et al. 2009; Li and Brüschweiler 2012, 2015; Meiler 2003; Shen and Bax 2007, 2010; Xu and Case 2001) and vice versa (Berjanskii et al. 2015; Shen and Bax 2015b; Shen et al. 2009), whereas in the context of IDPs these observables are primarily correlated with the protein sequence (Kjaergaard and Poulsen 2011; Schwarzinger et al. 2001; De Simone et al. 2009; Tamiola et al. 2010; Wang and Jardetzky 2002; Wishart et al. 1995). In the present study, we defined a method to generate CS tables in structured and disordered proteins by using exclusively the information contained in their sequences. This structure-free approach, protein sequences and chemical shift correlations (PROSECCO), was derived from the analysis of large experimental datasets by using a unique statistical approach that in the case of IDPs enabled to improve the state of the art of the prediction of CS and in the case of structured proteins achieved the accuracy levels of powerful structure-based methods. In addition to providing a tool for predicting CS using as input exclusively the protein sequence, the parameterization of PROSECCO has revealed key insights into the structural dependences of protein chemical shifts.

## Results

We used sequence homology criteria to select a database of non-redundant proteins whose chemical shifts were deposited in the biological magnetic resonance data bank (BMRB) (Ulrich et al. 2008) (BMRB, see “Materials and methods”). In total, 20,154 experimental chemical shifts of atoms from disordered proteins were used to derive a CS predictor for IDPs—PROSECCO_*IDP*_—and 3,953,878 experimental data were employed to generate a sequence-based CS predictor for structured proteins—PROSECCO_*FOLDED*_—(see “Materials and methods” and supplementary Tables S1–S3).

### Gaussian kernel-based neighbor correction in CS prediction of IDPs

We previously showed that loop regions of natively folded proteins are excellent models to describe the CS of random coil protein states (De Simone et al. 2009). In defining PROSECCO_*IDP*,_ we here identified a different approach that is based on the analysis of experimental CS from a pool of disordered proteins and on the use Gaussian kernel functions to generate density probability functions, $\widehat {d}_{i}^{A}\left( \delta \right)$:

1 $$\widehat {d}_{i}^{A}(\delta )=\frac{1}{{n_{i}^{A}}}\sum\limits_{{l=1}}^{{n_{i}^{A}}} {{G_K}(\delta - {\delta _l})}$$

In particular, for an atom of type *A* of an amino acid of type *i*, a specific probability density function $\widehat {d}_{i}^{A}\left( \delta \right)$ was generated by summing $n_{i}^{A}$ Gaussian kernels centered at the CS values of $n_{i}^{A}$ experimental observations (Fig. S1). The density function provides an expectation value for the CS that, depending on the amount of statistics, is evaluated as the weighted δ in $\widehat {d}_{i}^{A}\left( \delta \right)$ or to the δ associated with highest probability in the function (Fig. S1).

In addition to the primary term of prediction, we introduced residue pair-wise correction terms that incorporate the effects of the local sequence in a window of five residues. In particular, these correction terms were derived from pair-wise density functions $\widehat {d}_{{i,j}}^{A}\left( \delta \right)$ (Fig. S2) corresponding to sub-datasets of our database featuring the specific pair of amino acids i and j of the input sequence.

2 $$\widehat {d}_{{i,j}}^{A}(\delta )=\frac{1}{{n_{{i,j}}^{A}}}\sum\limits_{{l=1}}^{{n_{{i,j}}^{A}}} {{G_K}(\delta - {\delta _l})}$$

The expectation values of the pair-wise density functions, $\delta _{{i,j}}^{A}$, are therefore used to calculate the corrections to the primary term of chemical shift prediction, $\Delta \delta _{{i,~j}}^{A}=~\delta _{{i,j}}^{A} - \delta _{i}^{A}$. In the cases of pairs of residues with limited statistics in the database (i.e. < 20 observations), averaged nearest-neighbor correction terms were calculated $\Delta \delta _{{i,j}}^{A}$, corresponding to the overall effect that the amino-acid of type j exerts on all types of neighbor amino-acids in the position of the residue i.

The weights of the pair-wise correction terms were evaluated using the negative overlap between the primary and pair-wise density functions:

3 $$w=\left( {1 - \int {\hbox{min} \;(\widehat {d}_{i}^{A}(\delta ),\widehat {d}_{{i,j}}^{A}(\delta )} )d\delta } \right)$$

Finally, an overall normalization factor—*N*_*W*_—was introduced to balance the contribution of the primary term of CS prediction and the nearest-neighbor corrections. *N*_*W*_ values were calibrated for each atom by maximizing the agreement between the calculated and experimental CS (Fig. S3).

Taken together, these terms define PROSECCO_*IDP*_, a method using the information of the protein sequence to calculate CS of disordered proteins. In particular, in the case of the atom *A* of the residue *i* in a local sequence *k-j-* *i* *-l-m*, the overall equation of PROSECCO_*IDP*_ is:

4 $$CS_{i}^{A}=\delta _{i}^{A}+\frac{1}{{{N_W}}}\left( {w_{{i,{k^{ - 2}}}}^{A}\Delta \delta _{{i,{k^{ - 2}}}}^{A}+w_{{i,{j^{ - 1}}}}^{A}\Delta \delta _{{i,{j^{ - 1}}}}^{A}+w_{{i,{l^{+1}}}}^{A}\Delta \delta _{{i,{l^{+1}}}}^{A}+w_{{i,{m^{+2}}}}^{A}\Delta \delta _{{i,{m^{+2}}}}^{A}} \right)$$

**Fig. 1:**
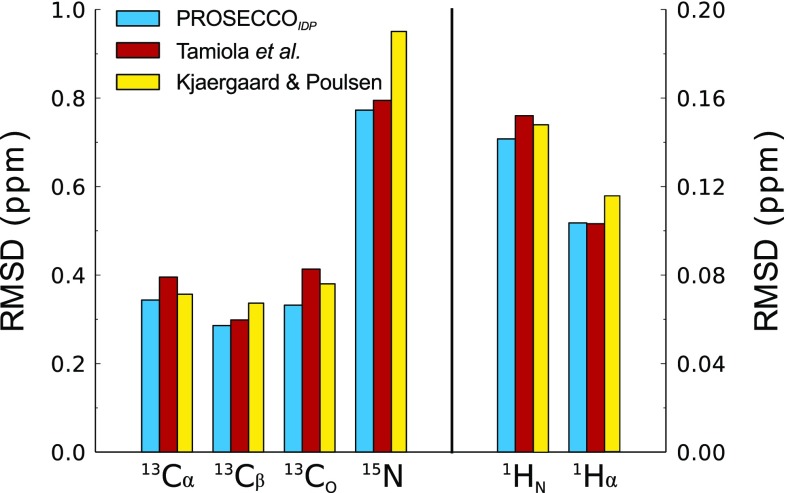
Sequence-based prediction of CS in IDPs. Root mean square deviations (RMSDs) are reported between experimental and predicted CS using PROSECCO_*IDP*_ (cyan) and the methods by Tamiola et al. (2010) (red) and Kjaergaard and Poulsen (2011) (yellow). In the case of PROSECCO_*IDP*_, the benchmark was performed using a “leave-one-out” approach, whereby when a BMRB entry is used to calculate the RMSD between experimental and calculated CS, the method is reparameterized by excluding this entry from the parameterizing dataset. The leave-one-out benchmark has been rotated on all the BMRB entries employed in PROSECCO_*IDP*_. In the two other programs tested, the benchmark was performed on the whole dataset of BMRB entries used in PROSECCO_*IDP*_. A web server for the PROSECCO method is available at http://desimone.bio.ic.ac.uk/prosecco/

We compared the performance of the method described in our study with that of the current most accurate predictors of CS of IDPs by Tamiola et al. (2010) and by Kjaergaard and Poulsen (2011) (Fig. 1). The benchmark indicates that PROSECCO_*IDP*_ improves the overall prediction of CS in disordered proteins, with particular accuracy in the case of backbone carbon atoms, which are sensitive probes of the local secondary structures in proteins (Camilloni et al. 2012a; Maltsev et al. 2012; Shen and Bax 2012).

### Prediction of chemical shifts in secondary structure elements of natively folded proteins

**Fig. 2:**
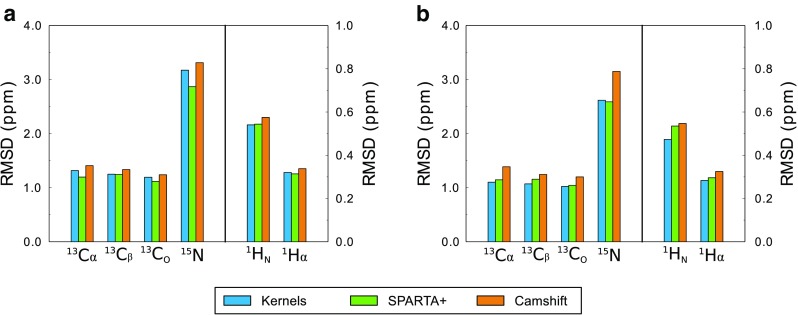
Gaussian-kernel prediction of chemical shifts in folded proteins classified in Q3 regions. The benchmark compares the performance of structure-based methods such as SPARTA+ (Shen and Bax 2010) and CamShift (Kohlhoff et al. 2009) with the prediction of CS using the Gaussian-kernels in indexed Q3 regions (helices, strands and coils) of the protein sequence. The dataset for this benchmark included 77 BMRB entries of structured proteins that were deposited from 2016 onwards (see Table S4 for the list BMRB entries and the corresponding PDB codes). **a** Benchmark performed including the whole protein sequences. **b** Benchmark performed by discarding two residues from each termini of the Q3 regions. A dissection of the accuracy in the different Q3 types is reported in Fig. S4

We then extended the kernel-based approach to the prediction of chemical shifts of secondary structure elements in natively folded proteins. To this end, we analyzed a database of nearly four millions CS of structured proteins to generate density probability functions and pair-wise correction terms for three types of structural elements, namely helixes (combined α-helixes and 3_10_-helixes), β-strands (combining both parallel and antiparallel β-sheets) and coils, in analogy with the Q3 classification that is common to many predictors of protein secondary structure. The resulting sequence-based prediction in the Q3 segments of folded proteins was benchmarked against a database containing 77 BMRB entries that were not included in the training dataset (“Materials and methods” and Table S4). The results showed a level of accuracy in the sequence-based CS prediction that is close to two powerful methods exploiting the analysis of protein three-dimensional structures, namely SPARTA+ (Shen and Bax 2010) and Camshift (Kohlhoff et al. 2009) (Fig. 2a). A higher accuracy was found when the benchmark of the sequence-based prediction excluded two residues for each N- and C- end of the Q3 regions, resulting in RMSD values that are similar to those obtained with SPARTA+ (Shen and Bax 2010) and better than those associated with Camshift (Kohlhoff et al. 2009) (Fig. 2b). This finding indicates that the protein sequence is the dominant factor in determining the CS values in internal regions of Q3 segments, but also evidenced that boundary regions between different Q3 segments require further terms of refinement to optimize the sequence-based CS prediction.

**Fig. 3:**
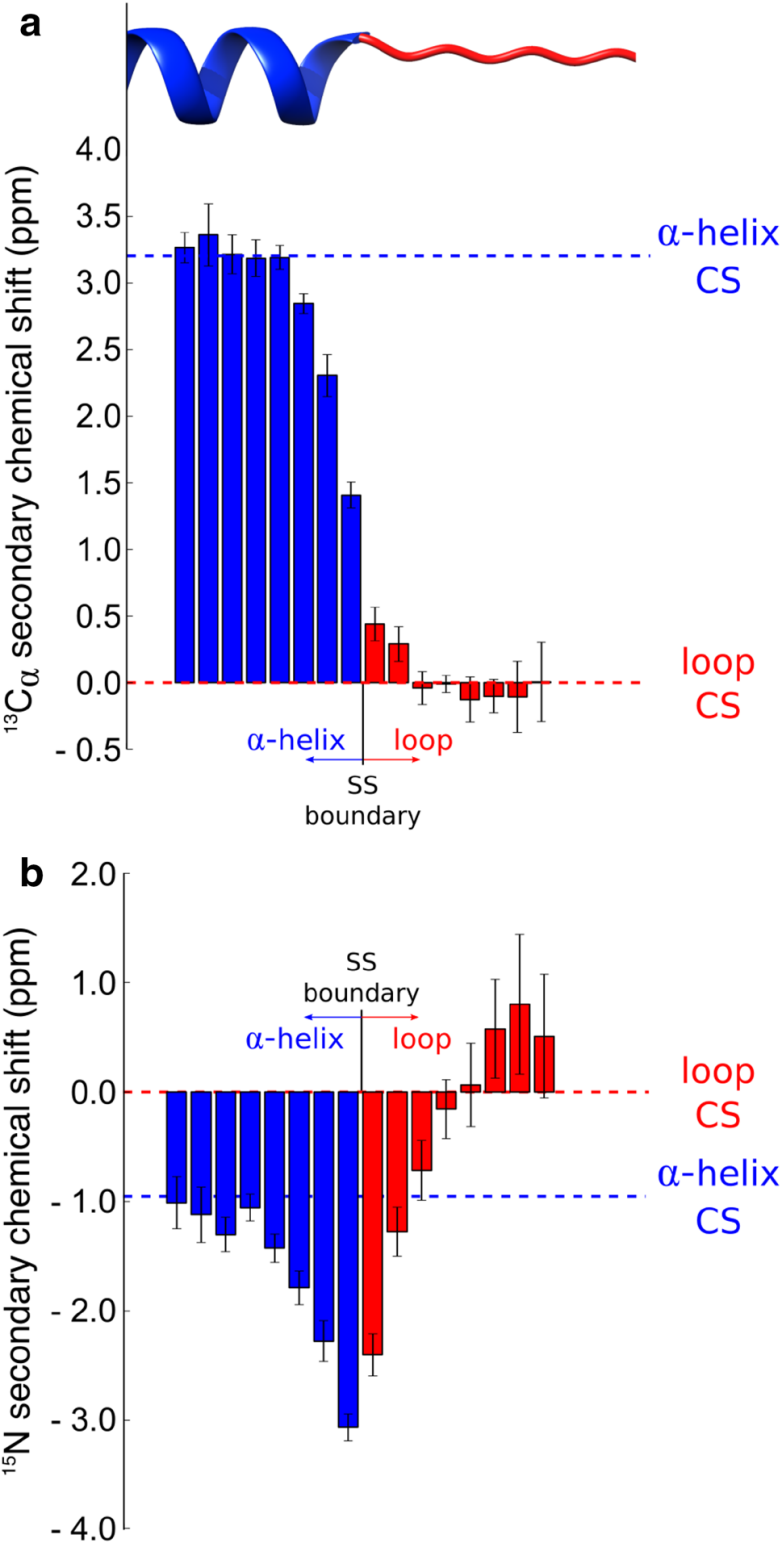
Secondary shifts in boundary regions between Q3 segments. The example of the boundary region between α-helixes and loops is shown. Bars report the average secondary shifts as a function of the distance from the boundary between the two Q3 segments, with error bars showing the standard deviations. **a** ^13^Cα secondary shifts gradually morph from the typical values adopted in α-helixes (+ 3.2 ppm) to those of loop regions (0.0 ppm). **b** Backbone amide ^15^N secondary shifts, however, exhibits anomalous trends. Starting from the typical values of α-helixes (− 0.95 ppm), the secondary shifts augment to a maximum value of − 3.0 ppm in correspondence of the last residue of the α-helixes to subsequently inverting toward positive values, with a maximum reached at the position 6 of the loop, and to finally fading to 0.0 ppm

To identify an improved approach for treating the boundary regions, we calculated the distributions of the secondary shifts along the protein sequences, which are defined by the difference between experimental and the random coil CS. This analysis indicated that, in the boundary across two Q3 regions, the secondary shifts gradually morph from those of one segment into the characteristic values of the following (Fig. 3a). Some anomalous transitions, however, were observed such as for example the backbone amide ^15^N secondary shifts in the boundary region between helixes and coils (Fig. 3b). Overall, this analysis identified specific patterns of secondary shifts in the boundary regions between Q3 segments, which enabled to generate ad hoc corrections terms resulting in an improved performance of 7.7% in the boundary regions, with an overall improvement of 3.6% (Fig. S5).

### An accurate sequence-based CS predictor for structured proteins

**Fig. 4:**
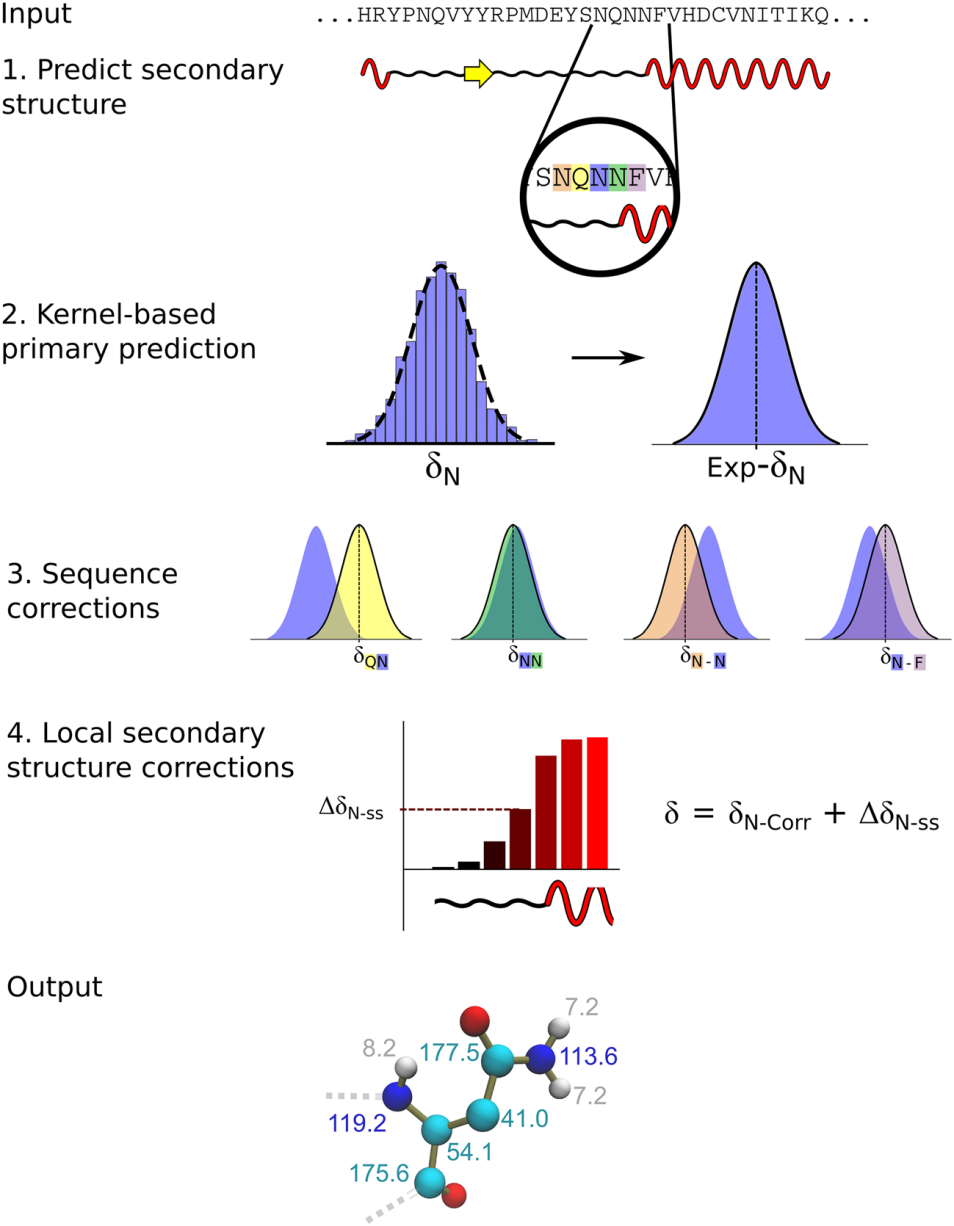
PROSECCO_*FOLDED*_ Scheme of the structure-free prediction of PROSECCO_*FOLDED*_ As example, a local segment of sequence “NQNNF” is used to illustrate how the prediction of the chemical shifts of the atoms in the central asparagine is generated. In the step 1, the protein sequence is analyzed using psipred (Jones 1999) to predict the secondary structure profile that provides the estimation of the Q3 regions (helixes, strands and coils) of the protein. In steps 2 and 3 the kernel-based prediction is applied to obtain CS tables in each of the Q3 segments, including the corrections of the boundary regions (step 4). The combination of all the prediction terms generates the output CS values (step 5)

The results of the kernel-based prediction in the Q3 segments indicated that an entirely sequence-based method to predict CS in folded proteins is possible, providing that the Q3 regions of the target proteins are known. Indeed in the above benchmarks, the Q3 regions were indexed from the analysis of the protein structures by DSSP (Kabsch and Sander 1983), however, the significant progresses made in the field of the secondary structure predictions enabled us to define a prediction of CS that is completely independent from experimental protein structures (Fig. 4). In this method, the kernel-based approach to generate CS tables is coupled with an estimation of the Q3 regions along the sequence by means of psipred (Jones 1999). The benchmark of this completely structure-free approach indicates that the uncertainty associated with the secondary structure prediction results in a minimal increase of RMSD values between calculated and experimental CS (only 5.5% compared to the case in which Q3 regions are identified from the experimental protein structures, Fig. S6). Remarkably, despite the such deterioration of performance, the sequence-based method was found to be more accurate than a structure-based predictor such as CamShift (Kohlhoff et al. 2009), although ultimately its performance resulted worse than that of SPARTA+ (Shen and Bax 2010) (Fig S6).

In order to minimize the error introduced when Q3 regions are estimated using psipred (Jones 1999), we introduced an artificial neural network. This network, which employs the information of the local sequences and local secondary structures, is defined with a single hidden layer and a single-node output layer, corresponding to the predicted CS values (see “Materials and methods”). Our benchmark shows that the network was successful in minimizing the difference in accuracy of the sequence-based prediction applied by using Q3 regions estimated with psipred or Q3 regions derived from the experimental protein structures (1.6% difference in RMSD values, Fig. S7).

**Fig. 5:**
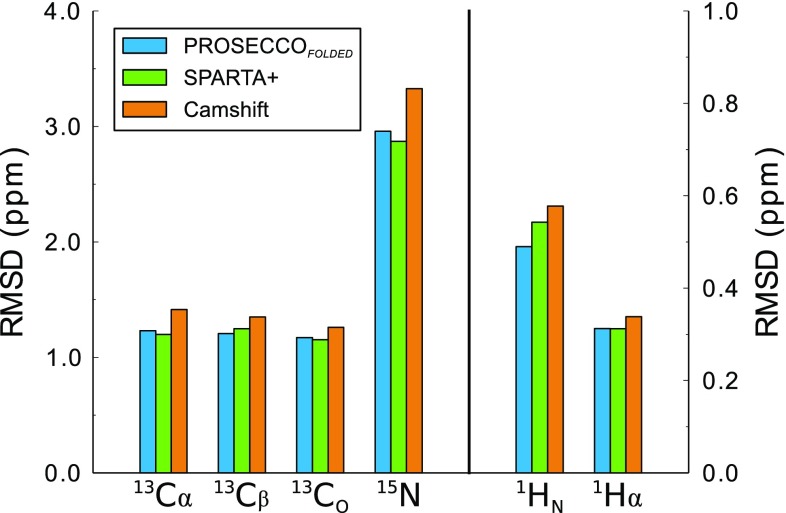
Accuracy of PROSECCO_*FOLDED*_ coupled with an artificial neural network. In its final version, PROSECCO_*FOLDED*_ was coupled with an artificial neural network to minimize the uncertainty introduced by using predicted secondary structure elements to index the Q3 regions. RMSD values between experimental and calculated CS showed that the sequence-based approach defined in this way reached a similar accuracy of predictors relying on structural similarity criteria such as SPARTA+ (Shen and Bax 2010) and a better performance than methods employing first principle approaches to analyze protein structures such as Camshift (Kohlhoff et al. 2009)

Taken together, these analyses define a purely sequence-based method, PROSECCO_*FOLDED*_, that is able to predict CS in folded proteins with a similar accuracy than methods exploiting structural-similarity criteria, SPARTA+ (Shen and Bax 2010), and higher accuracy than methods using first-principle analyses of the protein structures, Camshift (Kohlhoff et al. 2009) (Fig. 5). The excellent RMSD values for the sequence-based prediction of PROSECCO_*FOLDED*_ are also reflected in the correlations showed in the scatter plots (Fig. S8).

While the benchmark on the whole database suggests that PROSECCO_*FOLDED*_ can reach similar accuracy than SPARTA+ (Fig. 5), the case-by-case comparisons indicate that some proteins are better predicted than others (Fig. S9). More specifically, we found that in general PROSECCO_*FOLDED*_ has better performances with proteins rich in α-helix than β-sheet rich systems, a finding that reflects the performance of PROSECCO in the specific secondary structure elements (Fig. S4).

It is worth noting that the success of the sequence-based prediction by PROSECCO_*FOLDED*_ depends on the quality of the secondary structure prediction that generates the Q3 indexing. While the accuracy of the current alghoritms such as psipred is generally extremely high, some isolated cases are known where the quality of the secondary structure prediction is low. In these cases, the accuracy of the chemical shift prediction by PROSECCO_*FOLDED*_ can be severely affected. We illustrate this point using the example of the GA^95^/GB^95^ proteins, two systems designed to have 95% sequence identity but to fold into very different protein topologies (Shen et al. 2009). The secondary structure prediction of these two proteins, which differ for only three residues of their sequences, is extremely challenging for the secondary structure predictors, including psipred. When using PROSECCO_*FOLDED*_ to predict the chemical shifts of GA^95^/GB^95^, the error associated with psipred, which predicts essentially the same incorrect secondary structure pattern in both proteins (Fig. S10), is propagated to the chemical shift prediction, resulting in worse performance than SPARTA+ (Fig. S10). However, if the correct secondary structure pattern is provided, PROSECCO_*FOLDED*_ generates highly accurate chemical shifts with a similar precision to SPARTA+.

### Extending the prediction to side chains and different experimental conditions

The large datasets of chemical shifts employed in this investigation enabled a variety of atom types and experimental conditions to be parameterized. In particular, in addition to backbone atoms, PROSECCO generates accurate CS of side chain atoms (Table S5). Among these CS, a particular relevance is given to methyl groups, as these are accurate probes of structure and dynamics of large structured proteins via TROSY experiments (Ollerenshaw et al. 2003). The prediction of chemical shifts of methyl groups by PROSECCO resulted more accurate in the case of IDPs than in the case of folded proteins (Fig. S11a), which for the second is likely due to the lack of information on the tertiary structure, including effects such as the current ring shifts that strongly affects the CS of methyl protons. When compared with structure-based methods such as CH3SHIFT (Sahakyan et al. 2011) (Fig. S11b), the sequence-based prediction of methyl CS in PROSECCO_*FOLDED*_ resulted more accurate in the case of ^13^C atoms (3.5% lower RMSD values) and only 9.5% less accurate in the case of ^1^H atoms, which is remarkable considering that the CS of these atoms are significantly influenced by the protein tertiary structure.

Other features of PROSECCO include the ability to distinguish between oxidized and reduced cysteine residues, *cis*- and *trans*-proline residues, and protonated and non-protonated histidine residues. Finally, the distribution of pH values in the CS databases enabled to calibrate two different pH values (Fig. S12) in PROSECCO, namely 6.4 and 2.8.

## Discussion

The approach that we have illustrated has the unique ability to characterize NMR chemical shifts in both structured and disordered proteins using exclusively the information contained in their amino acid sequences. The statistical approach generating PROSECCO has taken advantage of the information contained in large datasets of experimental chemical shifts, which enabled a variety of experimental conditions and system properties to be parameterized, including two types of native states of proteins (folded and intrinsically disordered), two pH values (6.4 and 2.8), main chain and side chain atoms, *cis* and *trans* proline residues and oxidized/reduced cysteine residues. In predicting CS of IDPs, PROSECCO improves the accuracy of the current sequence-based predictors, whereas in the study of folded proteins it achieves similar levels of accuracy of SPARTA+ (Shen and Bax 2010) and an overall higher precision than Camshift (Kohlhoff et al. 2009), but in contrast to these methods without exploiting the information contained protein three-dimensional structures.

In addition to providing a sequence-based tool for treating chemical shifts of both structured and IDPs, the analyses leading to PROSECCO have revealed some key structural dependencies of protein chemical shifts. In particular, they showed that the backbone chemical shifts of residues in internal regions of Q3 segments are mostly independent from the tertiary and quaternary structure of the protein, and rely mainly on the local sequences. Moreover, the boundaries between Q3 segments were shown to adopt secondary shifts that morph between the values of adjacent segments, but in some cases anomalous patterns, such as for example the transition between helix and coil regions, reveal insights to account in structure-based CS predictors as well as methods to generate CS restraints to guide structural refinements in proteins.

## Conclusions

In conclusion, our data show that the sequence-based prediction of chemical shifts in folded proteins can be as accurate as that achieved by structure-based methods, and this feature enables to define a method for treating both structured and disordered proteins. As NMR is assuming a primary role in characterizing the structure and dynamics of proteins that cannot be studied using other approaches of structural biology, and for which often there is no structure available, an accurate sequence-based prediction of CS will support a large number of NMR investigations. The ability to treat with the same server both structured and disordered proteins is also convenient in the study of proteins that possess both types of characters, and for which current programs can only account either of the folded or of the disordered regions. Our results also suggest that the prediction of chemical shifts in biomacromolecules will be further improved by exploiting the increasingly growing information contained in databases of NMR chemical shifts. The PROSECCO method is available as a web server at http://desimone.bio.ic.ac.uk/prosecco/.

## Materials and methods

### Construction of the database of chemical shifts of proteins

In this investigation we employed an initial dataset of 9514 chemical shift assignments of proteins in the BMRB (Ulrich et al. 2008). This database was subjected to a series of filtering steps to remove sequence redundancies and poorly referenced entries. In particular, using the USEARCH algorithm (Edgar 2010), the initial database was filtered to ensure a maximum sequence identity between protein entries of 90%, which under these criteria resulted in 5140 non-redundant protein entries. The dataset selection was further refined by discarding the entries that showed an incorrect referencing, as assessed by comparing individual chemical shifts with the overall distribution in the BMRB for the specific type of atom and amino acid in the associated secondary structure element. Using this benchmark, entries that had overall CS deviations from the expected value (the center of the distribution) of more than δ^tolerance^ (tolerance values of 1.5, 0.5 and 3.5 ppm for ^13^C, ^1^H and ^15^N respectively) were deemed as incorrectly referenced. The referencing analysis resulted in the removal of 147 BMRB entries. Additionally, specific CS values in otherwise well-referenced BMRB entries were removed if deviating of more than 3.5 standard deviations from their expected values (center of the corresponding distribution), amounting to a total of 0.51% values of the dataset.

### Dataset for disordered proteins

Forty-four entries of our CS database were from IDP or proteins containing an intrinsically disordered region (IDR) of at least 20 residues. IDPs and IDRs were identified by cross-referencing with Disprot (Piovesan et al. 2017) or specific annotations in the literature. The resulting database contained a total of 20,154 chemical shifts from IDPs or IDRs, which were used to parameterize PROSECCO_*IDP*_. To avoid reductions in the number of experimental data, the whole set of CS was employed in the parameterization, and the *leave-one-out* approach was used to compute the RMSDs between experimental and calculated CS during the benchmarks as previously done (De Simone et al. 2009). A list of BMRB codes for the database employed in the parameterization of PROSECCO_*IDP*_ is provided in the supplementary materials (Table S1).

### Dataset for folded proteins

The final version of the database of CS contained 4993 BMRB entries of structured proteins, which resulted in 3,953,878 experimental data composing the parameterizing dataset for PROSECCO_*FOLDED*_. Within this database, however, only 2943 entries were associated with a protein structure in the protein data bank (PDB), which flags the importance of sequence-based CS predictors such as PROSECCO. As a result, in order to assign the regions of helixes, strands and coil (the Q3 segments) for the whole set of 4993 entries, we employed the δ2D (Camilloni et al. 2012b), which is an accurate method to individuate secondary structure elements from protein chemical shifts. Additional entries to the parameterizing dataset were employed exclusively for benchmarking PROSECCO_*FOLDED*_, SPARTA+ (Shen and Bax 2010) and CamShift (Kohlhoff et al. 2009). In particular, the selected benchmark database contained 77 BMRB entries having all been deposited after 2016, which ensured that these data were not part of the parameterization of SPARTA+ (Shen and Bax 2010) and CamShift (Kohlhoff et al. 2009). The lists of BMRB codes composing the databases employed for the parameterization (Tables S2 and S3) and the benchmarks (Table S4) of PROSECCO_*FOLDED*_ are provided in the supplementary materials.

### Neural network architecture

The final implementation of PROSECCO_*FOLDED*_ included a feedforward single-layer regressor neural network model composed of 143 input nodes. This neural network has proved to be a powerful tool in a variety of prediction algorithms across a spectrum of scientific disciplines (Papik et al. 1998; Zupan and Gasteiger 1999), and have also been previously employed in the prediction of chemical shifts based from three-dimensional structures (Li and Brüschweiler 2012; Meiler 2003; Shen and Bax 2010).

In particular, we used a neural structure of a directed mathematical graph, where an initial layer of input data is coded by assigning the data on individual nodes of the graph. Our inputs include local sequence and local secondary structure (Q3 classification of helixes, strands and coils). The neural network therefore converts the nodes from the input layer into a single output value, the predicted chemical shift, via a series of mathematical operations. The first step is to connect the input layer with a hidden layer of nodes, where hidden nodes store the results of the weighted sum of input nodes. The weights of these sums have been calibrated during the training of the network to model complex relationships between the inputs and the output (Hornik 1991). The calibration revealed an optimal amount of nodes in the hidden layer of 50. In the second step, the results of each hidden node are transformed using an activation function, typically a sigmoid or a hyperbolic tangent function, to enable the algorithm to model nonlinear relationships between the data and the output variable. In our implementation, the hyperbolic tangent function generated more accurate results than sigmoid and rectified linear functions. Finally, a single output node, which contains the predicted chemical shift, receives the weighted sum of the hidden nodes, with weights that are also calibrated during the training of the network. The calibration of all the weights of the network was achieved by minimizing the mean squared error between the outputs and the experimental chemical shifts in the training dataset. The algorithm used for weight optimisation was Adam (Kingma and Ba 2015), a stochastic variation of the gradient descent algorithm, whereby the gradient of the mean squared error is evaluated at each step with respect to the weights, and these are then modified in order to minimize the error.

In the specific implementation of our network, we used 143 input nodes. These nodes include information for each residue in the sequence. In particular, four nodes were used to describe the secondary structure content (one node for each Q3 category and an extra node to account for terminal regions in the segments) in a window of seven residues of the protein sequence, amounting to a total of 28 nodes. The remaining 115 nodes describe the sequence effects on the chemical shifts by including a window of five residues (each residue described by 23 nodes: 20 standard amino acids and additional nodes for oxidized cysteines, *cis* prolines and protonated histidines). Instead of using binary values (0 or 1) on the 23 types of nodes describing the residue identities we employed the score from the BLOSUM-62 amino-acid similarity score matrix (Henikoff and Henikoff 1992), which increases the performance of the network as implemented in neural network employed in SPARTA+ (Shen and Bax 2010) and in other contexts (Berry et al. 2004; Lundegaard et al. 2011).

### Root mean square deviation (RMSD)

As a primary tool to compare the predicted and experimental chemical shifts, we used in our benchmarks the root mean square deviation between n calculated and experimental chemical shift values for a specific spin system:

5 $$RMSD=\sqrt {\frac{1}{n}\sum\limits_{{l=1}}^{n} {{{\left( {{\delta _{l,exp}} - {\delta _{l,calc}}} \right)}^2}} }$$

## Electronic supplementary material

Below is the link to the electronic supplementary material.

Supplementary material 1 (DOCX 8882 KB)

## Abbreviations

CS: Chemical shifts
IDPs: Intrinsically disordered proteins
IDRs: Intrinsically disordered regions
NMR: Nuclear magnetic resonance
PROSECCO: Protein sequences and chemical shift correlations
