# Supplementary material for: The PROSECCO server for chemical shift predictions in ordered and disordered proteins
Source: J Biomol NMR. 2017 Nov 8;69(3):147–56. doi: 10.1007/s10858-017-0145-2 (PMC5711976; doi:10.1007/s10858-017-0145-2)
Supplement: Supplementary file 1 — Supplementary material 1 (DOCX 8882 KB) [file 10858_2017_145_MOESM1_ESM.docx]

**A unified sequence-based approach to interpret NMR chemical shifts in ordered and disordered proteins**

Máximo Sanz-Hernández^1^ and Alfonso De Simone^1*^

^1^Deparment of Life Sciences, Imperial College London, SW7 2AZ, UK.

*Correspondence: adesimon@imperial.ac.uk

**Table of Contents:**

- Pages S1-S12: SI-Figures
- Pages S10-S28: SI-Tables
- Pages S29: SI-References

**SI-Figures**

**
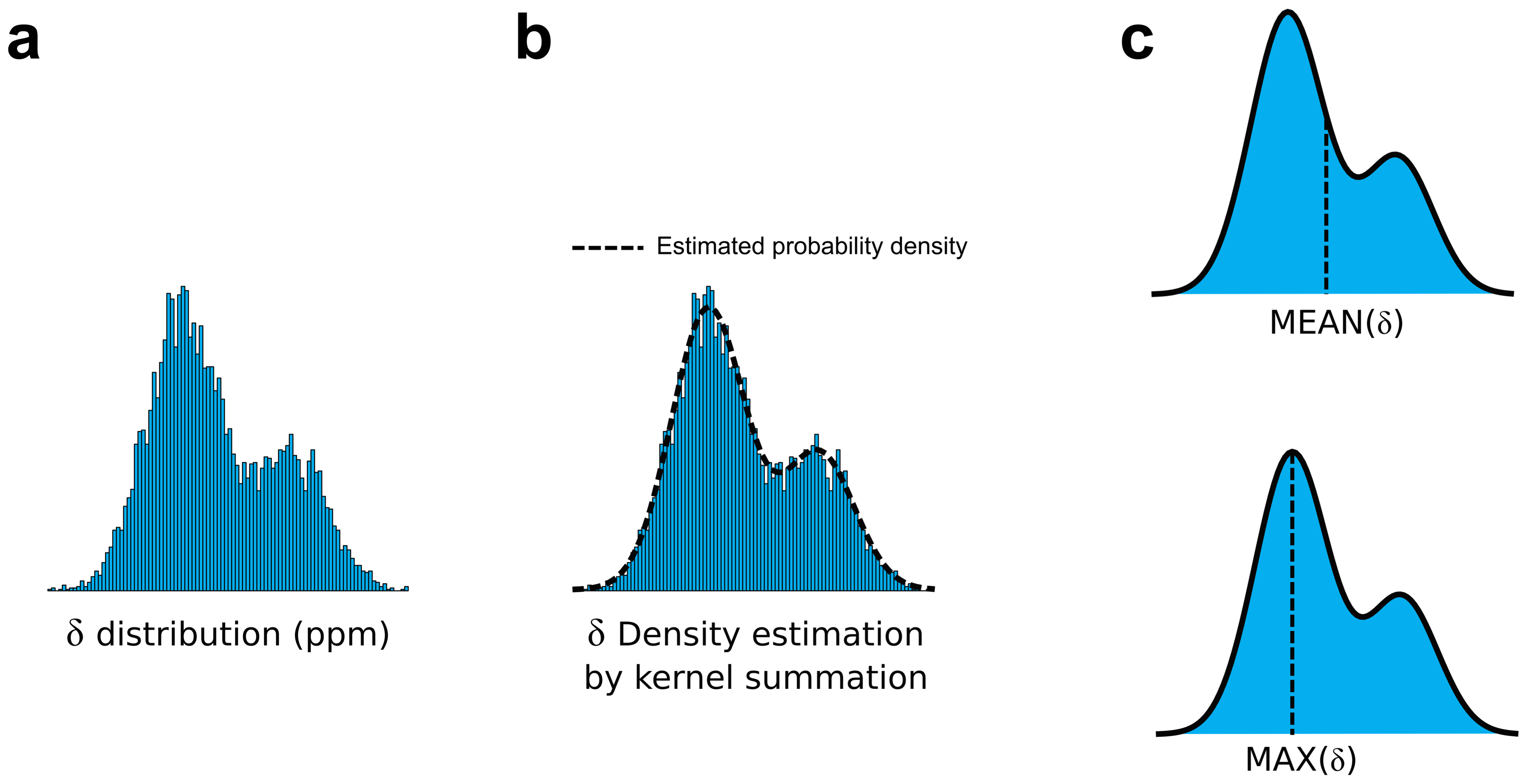
**

**Figure S1. Gaussian kernel sums providing probability density functions.** (a) Representative distribution of experimental chemical shifts, δ, for a specific spin system. The distributions are analyzed by summing Gaussian kernels centered on each experimental value. (b) The sum of kernels provides continuous probability density functions $\hat{d}_{i}^{A}\left( \delta\right)$ (dashed line). These functions are generally smooth in nature as the sum of the kernels minimizes spikes in the chemical shift distributions, a feature that provides great advantages in cases associated with poor statistics. (c) The expectancy value of the CS is evaluated either as the δ associated with the highest probability, $\delta_{i}^{A}=$max($\hat{d}_{i}^{A}\left( \delta\right)$), or as the average probability, $\delta_{i}^{A}=$E[$\hat{d}_{i}^{A}\left( \delta\right)$]. The first option is applied for distributions composed of less than 400 experimental data.

**Figure S2. Kernel-based pairwise neighbor corrections.** To account of local sequence effects, pair-wise correction terms were introduced. In particular, for a given atom of a specific amino-acid (in the schematic example an isoleucine residue, blue distribution), a subset of cases is identified in the CS database that matches the specific pair of residues featured in the input sequence (in the schematic example isoleucine-phenylalanine, yellow distribution). This subset is analyzed to generate a density probability function,$\hat{d}_{i,j}^{A}\left( \delta\right)$, that is specific for the pairs of amino-acids (eq. 2). The correction term is therefore calculated as the difference between the expectancy value of the overall density function and that of the pair-wise function, ${\Delta\delta}_{i, j}^{A}= {\delta_{i,j}^{A}-\delta}_{i}^{A}$. A crucial part of the correction is in its weighting, which is provided by the overlap between the two distributions (eq. 3) upon normalization (green areas in panels b and c).

**Figure S3. Calibration of the normalizing weight Nw.** The normalization factor Nw was introduced to provide an overall weight between the primary CS estimation and the nearest-neighbor correction terms. The example of the calibration curves for the amide ^15^N atom in PROSECCO*_IDP_* (a) and PROSECCO*_FOLDED_* (b) is shown. A single minimum in the RMSD was found in all cases, and individual Nw values were optimized for each atom in both PROSECCO*_IDP_* and PROSECCO*_FOLDED_*.


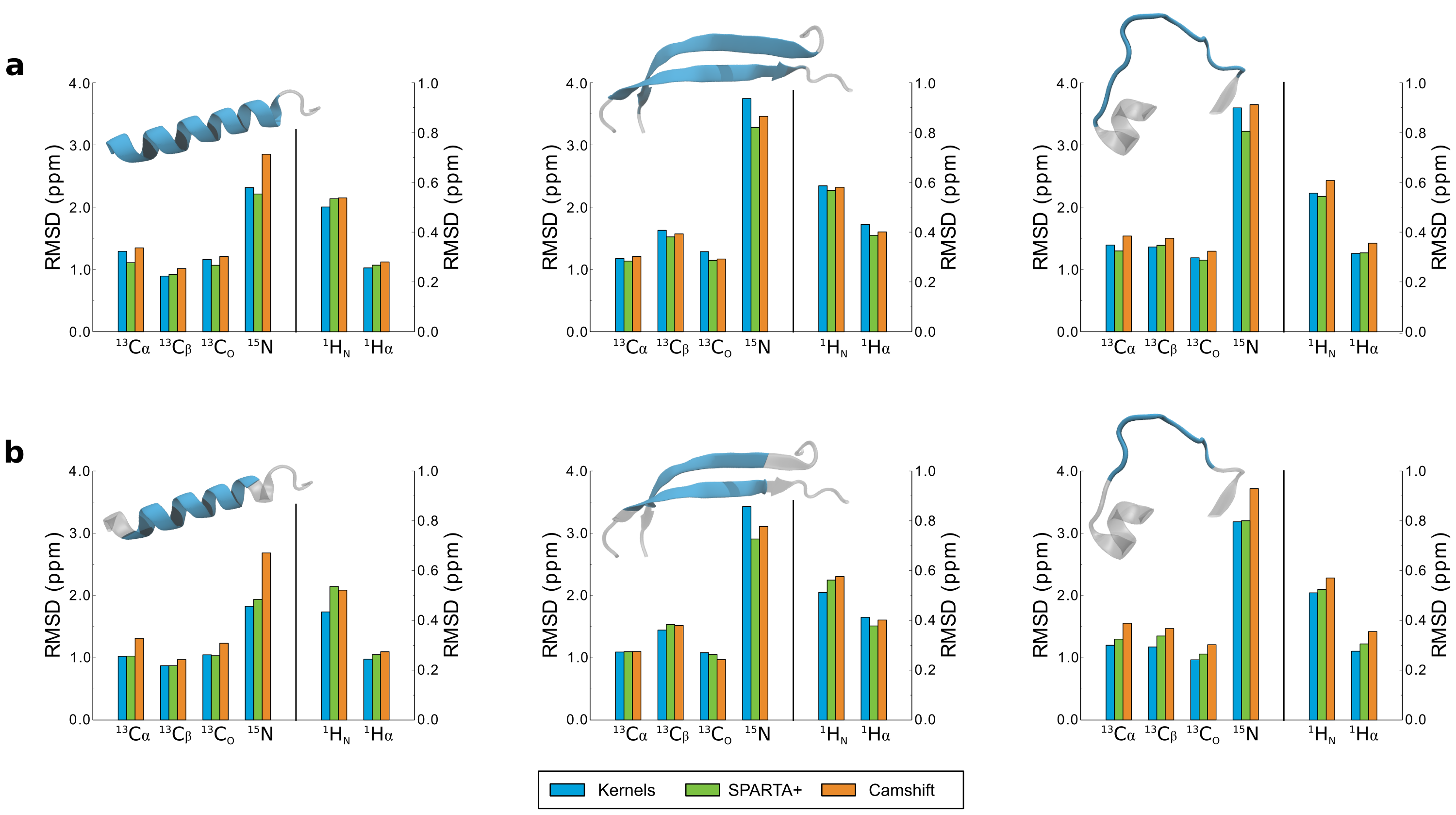


**Figure S4. RMSD values of the CS prediction in specific Q3 regions of folded proteins.** The dataset for this benchmark included 77 BMRB entries of structured proteins that were deposited from 2016 onwards (see Table S4 for the list BMRB entries and the corresponding PDB codes). (a) Benchmark performed including the whole protein sequences. (b) Benchmark performed by discarding 2 residues from each termini of the Q3 regions. The sequence-based kernels approach (cyan) is compared with the structure-based methods SPARTA+ (Shen and Bax, 2010) (green) and Camshift (Kohlhoff et al., 2009) (orange).

**Figure S5. Improvement generated by the correction terms in the boundary regions between Q3 segments.** The dataset for this benchmark included 77 BMRB entries of structured proteins that were deposited from 2016 onwards (see Table S4 for the list BMRB entries and the corresponding PDB codes). The sequence-based kernels approach applied with (violet) and without (cyan) corrections in the boundary regions between Q3 segments is compared with the structure-based methods SPARTA+ (Shen and Bax, 2010) (green) and Camshift (Kohlhoff et al., 2009) (orange). As a result of the correction terms in the boundary regions between Q3 segments, the sequence-based CS prediction improved of 3.6%, considering the whole protein sequences, with RMSD values reduced of 7.7% in the boundary regions.

**Figure S6. Performance of the kernels method coupled with psipred prediction of Q3 segments.** The structure-free method includes the estimation of the Q3 regions using psipred (Jones, 1999). This generates CS with RMSD values from experimental values that are only 5.5% higher than when the Q3 regions are indexed using the experimental protein structures. The dataset for this benchmark included 77 BMRB entries of structured proteins that were deposited from 2016 onwards (see Table S4 for the list BMRB entries and the corresponding PDB codes). The sequence-based kernels approach coupled with psipred (cyan) or applied by using PDB derived Q3 segments (violet) is compared with the structure-based methods SPARTA+ (Shen and Bax, 2010) (green) and Camshift (Kohlhoff et al., 2009) (orange).

**Figure S7. Benchmark of PROSECCO*_FOLDED_*.** The final version of PROSECCO*_FOLDED_* includes the application of a neural network to minimize the additional uncertainty introduced by using Q3 regions predicted using psipred (Jones, 1999). PROSECCO*_FOLDED_* coupled with psipred (cyan) generates CS with RMSD values from experimental values that are only 1.6% higher than when the method is applied using PDB derived Q3 segments (violet). The RMSD of structure-based methods SPARTA+ (Shen and Bax, 2010) (green) and Camshift (Kohlhoff et al., 2009) (orange) are also shown. The dataset for this benchmark included 77 BMRB entries of structured proteins that were deposited from 2016 onwards (see Table S4 for the list BMRB entries and the corresponding PDB codes).

**a**


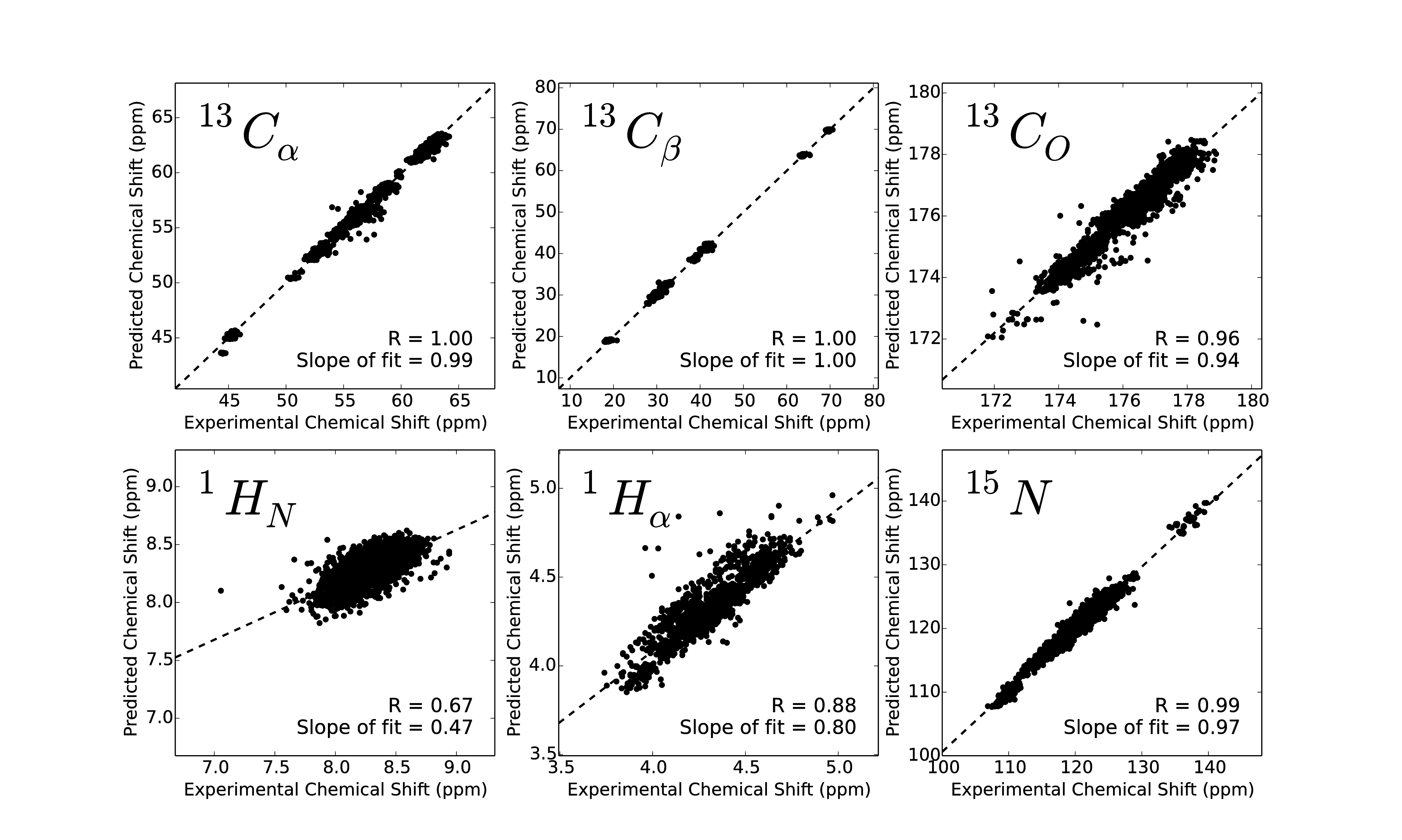


**b**


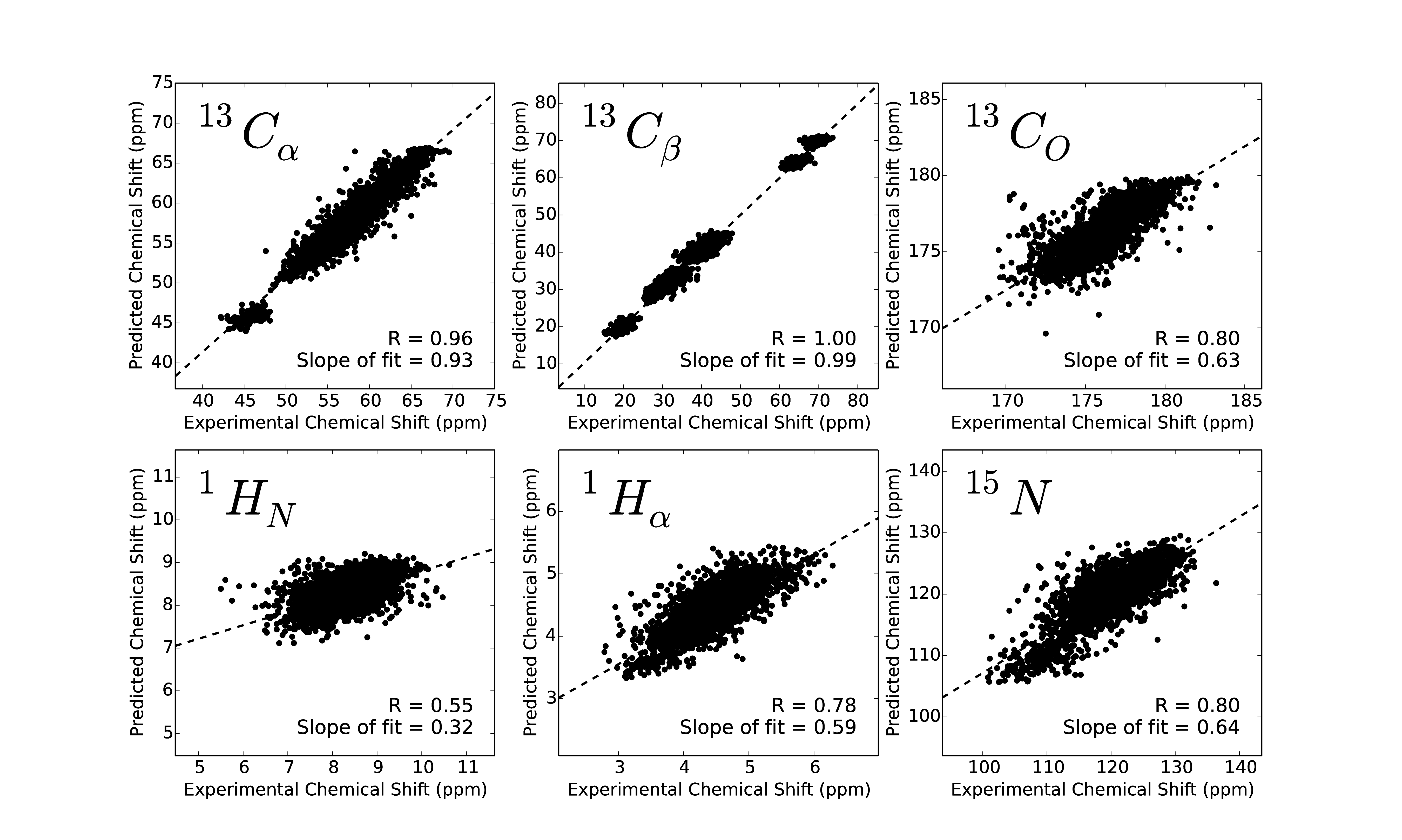


**Figure S8. Correlation plots and factors for the benchmark of PROSECCO for backbone atoms in (a) IDPs and (b) folded proteins.**


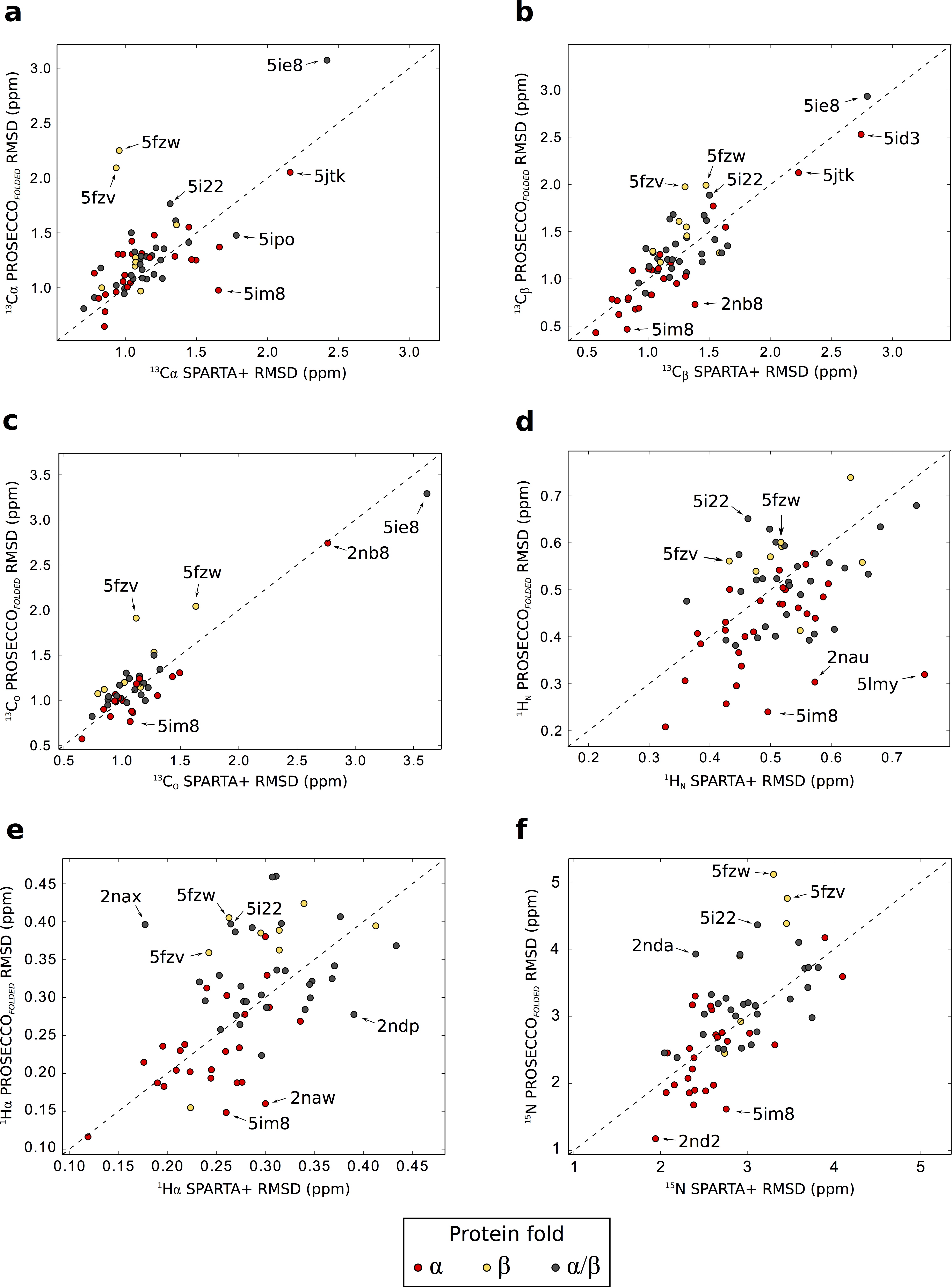


**Figure S9. RMSD values for individual BMRB entries from our benchmark on SPARTA+ and PROSECCO*_FOLDED_*.** Each point of this plot reports individual RMSD values of predictions made using PROSECCO*_FOLDED_* and SPARTA+ (Shen and Bax, 2010). Dots are colored to indicate the prevailing secondary structure content of the protein. PROSECCO*_FOLDED_* appeats to generally perform better with alpha-helical proteins and to have lower efficiency for proteins that are rich in beta-sheet structure, as compared with SPARTA+. BMRB entries employed in this benchmarks correspond to the following PDB codes: 2n41, 2n8z, 2n9z, 2naj, 2naq, 2nau, 2nav, 2naw, 2nax, 2nb1, 2nb5, 2nb6, 2nb8, 2nb9, 2nba, 2nbh, 2nbq, 2nbs, 2nc8, 2ncg, 2ncj, 2ncl, 2ncz, 2nd2, 2nd9, 2nda, 2ndf, 2ndn, 2ndp, 2rvq, 5frh, 5fzv, 5fzw, 5fzx, 5hp0, 5hpd, 5hv8, 5i1r, 5i22, 5id3, 5ie8, 5ieb, 5im8, 5ipo, 5ird, 5jpw, 5jtk, 5jtl, 5kes, 5kiz, 5knw, 5kp0, 5kpe, 5kph, 5krw, 5kvp, 5l3l, 5lam, 5lfi, 5lg9, 5lmy, 5lw8, 5m1h, 5mf9, 5mmu, 5t17, 5t3y, 5tbn. Some examples with different performance of the two programs are indicated using arrows and by reporting the corresponding PDB code.

**
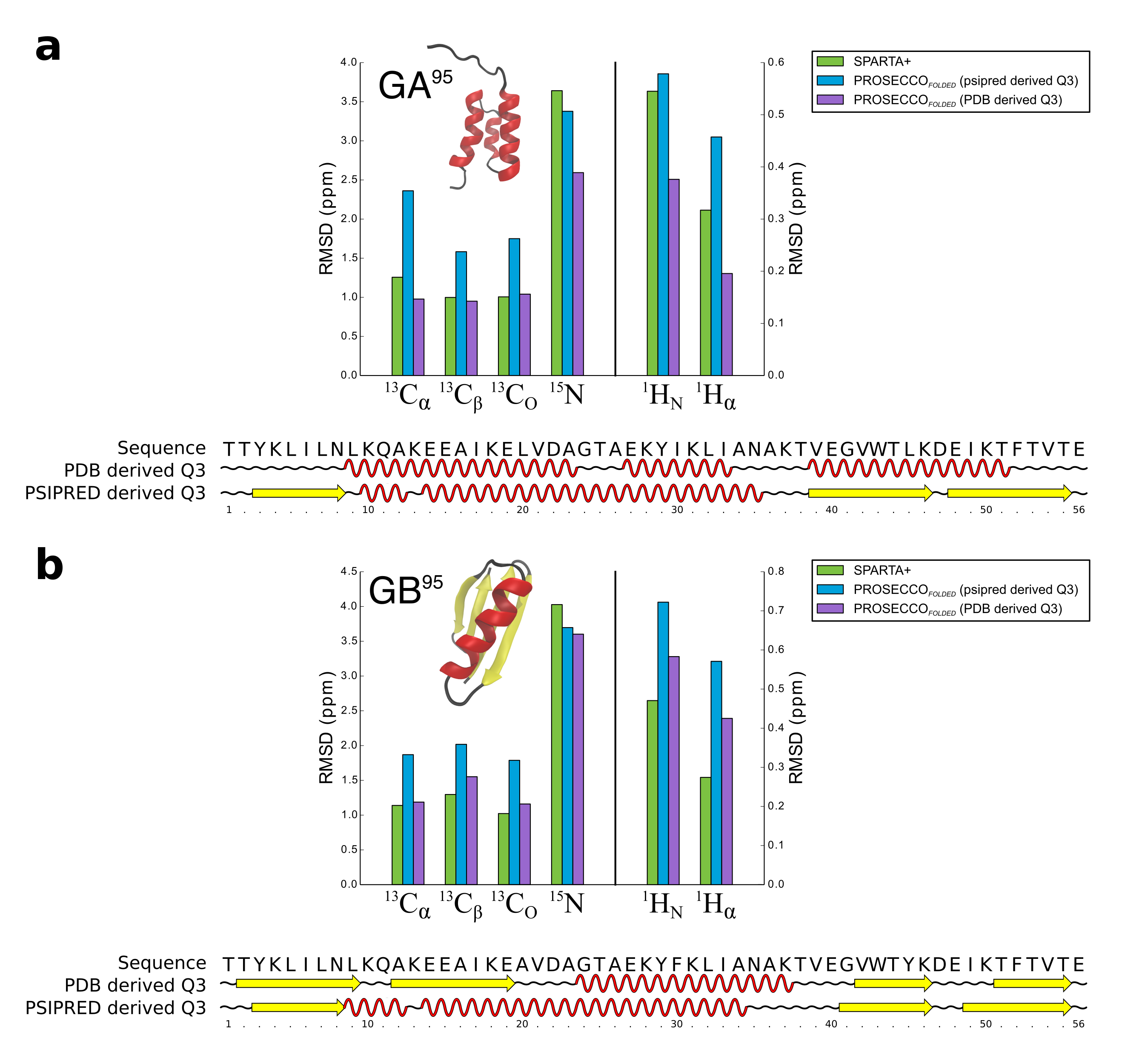
**

**Figure S10. Chemical shift prediction of the GA^95^/GB^95^ system (Shen et al., 2009).** Given their high sequence identity, psipred (Jones, 1999) predicts a virtually identical secondary structure for the two proteins, resulting in overall inaccurate chemical shift predictions. If the Q3 information provided to PROSECCO*_FOLDED_* is derived from the PDB structures instead, the predictions become very accurate, comparable to SPARTA+ (Shen and Bax, 2010) or even improving them in the case of the helical GA^95^.

**Figure S11. Prediction of methyl chemical shifts by PROSECCO*_IDP_* and PROSECCO*_FOLDED_*.** **a)** RMSD of the prediction of ^13^C and ^1^H chemical shifts of methyl groups in folded and intrinsically disordered proteins. **b)** Difference in the RMSD values for ^13^C and ^1^H chemical shifts of methyl groups in PROSECCO*_FOLDED_* and the structure-based method CH3SHIFT (Sahakyan et al., 2011).

**Figure S12.** **Calibration of low pH parameters in PROSECCO*_IDP_* and PROSECCO*_FOLDED_*.** The distribution of pHs in the parameterizing databases was primarily centered at pH 6.4 with a secondary peak at pH 2.8 corresponding to NMR investigations performed using low pH conditions. These included 158 and 6 BMRB entries in the cases of folded and disordered proteins, respectively. The calibration of pH sensitive residues was therefore performed for the low pH conditions by estimating the RMSD between calculated and experimental CS values as a function of a perturbation factor Δδ_i_. The example of calibration in PROSECCO*_IDP_* is provided.

**SI-Tables**

| **Table S1. BMRB codes of IDPs and IDRs used to parametrise PROSECCO*_IDP_***. | |
| --- | --- |
| **BMRB ID** | **DISORDERED SEGMENT** |
| 4286 | ALL (1 to 98) |
| 4108* | ALL (1 to 70) |
| 4375* | ALL (1 to 76) |
| 4676* | ALL (1 to 153) |
| 4716 | 6 to 85 |
| 5228 | 1 to 27 |
| 5335 | 43 to 68 |
| 5563 | 1 to 32 |
| 6055 | 1 to 20 |
| 6078 | 1 to 68 |
| 6098 | 1 to 59 |
| 6784 | ALL (1 to 60) |
| 6846 | 16 to 37 |
| 6968 | ALL (1 to 140) |
| 7244 | ALL (1 to 127) |
| 7358 | ALL (1 to 199) |
| 15123 | 1 to 36 |
| 15141 | 1 to 45; 52 to 74; 85 to 106 |
| 15179 | 2 to 92; 98 to 124; 139 to 159 |
| 15340 | 59 to 182 |
| 15397 | ALL (1 to 71) |
| 15409 | ALL (1 to 115) |
| 15430 | ALL (1 to 87) |
| 15441 | 41 to 67 |
| 15744 | 10 to 30; 176 to 200 |
| 15883* | ALL (1 to 92) |
| 16166 | 1 to 89 |
| 16296 | 14 to 85 |
| 16627* | ALL (1 to 56) |
| 16876 | 1 to 171 |
| 17205 | 1 to 26 |
| 17290 | 22 to 139 |
| 17499 | 1 to 22 |
| 17760 | 1 to 73 |
| 17920 | ALL (1 to 441) |
| 17926 | 39 to 81 |
| 18889 | ALL (1 to 57) |
| 18890 | ALL (1 to 46) |
| 19191 | ALL (1 to 70) |
| 19364 | 13 to 93 |
| 19723 | 39 to 99 |
| 25838 | 1 to 26; 31 to 101 |
| 26549 | 6 to 34; 71 to 119; 126 to 163; 177 to 268 |
| 26816* | ALL (1 to 160) |

*Entries used in the calibration at acidic pH

| **Table S2. BMRB and relative PDB codes of proteins associated with a three-dimensional structure, as used in the parameterization of PROSECCO*_FOLDED_***. | | | | | | | | | | | | | | | | | | | | | | | |
| --- | --- | --- | --- | --- | --- | --- | --- | --- | --- | --- | --- | --- | --- | --- | --- | --- | --- | --- | --- | --- | --- | --- | --- |
| **BMRB** | **PDB** | **BMRB** | **PDB** | **BMRB** | **PDB** | **BMRB** | **PDB** | **BMRB** | **PDB** | **BMRB** | **PDB** | **BMRB** | **PDB** | **BMRB** | **PDB** | **BMRB** | **PDB** | **BMRB** | **PDB** | **BMRB** | **PDB** | **BMRB** | **PDB** |
| 10002 | 1VEX | 10310 | 2YTK | 11348 | 2E63 | 15673 | 2K19 | 16561 | 2KPN | 17432 | 2L90 | 18329 | 2LQU | 19213 | 2M7U | 25098 | 2MRY | 4552 | 1G8C | 5598 | 1OV2 | 6454 | 2AVX |
| 10006 | 1WFU | 10312 | 2D9Z | 11349 | 1X4K | 15677 | 2K1E | 16562 | 2KPO | 17433 | 2L91 | 18332 | 2LQV | 19216 | 2M7X | 25102 | 2MS3 | 4553 | 1DGQ | 5601 | 1ZRP | 6464 | 1YEL |
| 10008 | 1WFT | 10313 | 2DA0 | 11350 | 1X4L | 15678 | 2K1H | 16563 | 2KPP | 17434 | 2L92 | 18338 | 2LUW | 19217 | 2M7Z | 25104 | 2MS4 | 4557 | 1QLO | 5604 | 1MWY | 6465 | 2AIZ |
| 10009 | 1WFW | 10314 | 2DHI | 11351 | 2YUE | 15679 | 2K1M | 16564 | 2KPQ | 17435 | 2L93 | 18341 | 4ASV | 19218 | 2M80 | 25122 | 2MSF | 4566 | 1L6U | 5610 | 1MV4 | 6475 | 2BGO |
| 10011 | 2COM | 10315 | 2DHJ | 11352 | 1WES | 15691 | 2K29 | 16570 | 2KPU | 17448 | 2L9D | 18345 | 2LR3 | 19219 | 2MR3 | 25127 | 2MSN | 4574 | 1D4B | 5617 | 1MVZ | 6479 | 1Y6U |
| 10013 | 1WIL | 10316 | 2DHK | 11353 | 1X4I | 15693 | 2K27 | 16572 | 2KPW | 17450 | 2L9G | 18346 | 2LR4 | 19225 | 2M83 | 25128 | 2MSO | 4577 | 1C05 | 5620 | 1NE3 | 6489* | 1WU0 |
| 10025 | 1UL7 | 10317 | 2DKP | 11354 | 1WEN | 15695 | 2K28 | 16576 | 2KQ1 | 17451 | 2L9J | 18347* | 2LR5 | 19229 | 2M85 | 25135 | 2MSV | 4583 | 1DOQ | 5621 | 1NEI | 6505 | 1YEZ |
| 10026 | 1V5S | 10318 | 2DKQ | 11355 | 2DK4 | 15701 | 2K2D | 16578 | 2KQ2 | 17478 | 2L9M | 18349 | 2LR6 | 19230 | 2M86 | 25137 | 2MSW | 4584 | 1DP3 | 5622 | 1NHO | 6506 | 2CA7 |
| 10027 | 2E0G | 10319 | 2DN6 | 11356 | 1UJY | 15702 | 2K2E | 16589 | 2KQ5 | 17481 | 2L9P | 18350 | 2LR7 | 19232 | 2M88 | 25139 | 2MSX | 4585 | 1DU9 | 5624 | 1ND9 | 6510 | 1YXE |
| 10028 | 2DWV | 10320 | 2DI9 | 11357 | 2DK7 | 15707 | 2K2J | 16592 | 2KQ8 | 17484 | 2L9R | 18351 | 4AOG | 19233 | 2MGP | 25147 | 2MT3 | 4587 | 1ED0 | 5631 | 1N7T | 6516 | 2FQ2 |
| 10029 | 1WFV | 10321 | 2DIB | 11363 | 1X4S | 15716 | 2ML1 | 16603 | 2KQK | 17488 | 2L9U | 18352 | 2LR8 | 19235 | 2M89 | 25148 | 2MT4 | 4589 | 1EHX | 5652* | 1NE5 | 6519 | 1YX8 |
| 10030 | 1WJO | 10322 | 2DIC | 11364 | 2DT6 | 15717 | 2LB9 | 16605 | 2KQL | 17493 | 2L9Y | 18354 | 2LR9 | 19237 | 2M8B | 25149 | 2MT5 | 4590 | 1EIG | 5657 | 1NR3 | 6520 | 2BIC |
| 10031 | 1WJR | 10323 | 2DJ4 | 11365 | 2DT7 | 15718 | 2K2O | 16610 | 2KQR | 17494 | 2L9Z | 18355 | 2LRA | 19249 | 2M8E | 25150 | 2MT6 | 4591 | 1EJP | 5664 | 1NQ4 | 6529 | 2LVF |
| 10032 | 1WJS | 10325 | 2DMB | 11366 | 2FHO | 15721 | 2N4D | 16617 | 2KQY | 17500 | 2LA2 | 18356 | 2MP4 | 19251 | 2M8G | 25151 | 2MT7 | 4603 | 1DV5 | 5667 | 1N0Z | 6530 | 1YMZ |
| 10033 | 1WJZ | 10326 | 2DMC | 11368 | 2YSO | 15723 | 2K87 | 16620 | 2KR1 | 17501 | 2LA3 | 18357 | 2LRD | 19252 | 2M8H | 25152 | 2MT8 | 4607 | 1EQX | 5669 | 1NJ3 | 6536 | 2GTJ |
| 10034 | 1WK0 | 10327 | 2DS4 | 11369 | 2YSP | 15734 | 2K31 | 16624 | 2KR5 | 17502 | 2LA4 | 18374 | 2LRI | 19274 | 2M8U | 25153 | 2MT9 | 4615 | 1DKC | 5674 | 1NIY | 6540 | 2AXL |
| 10035 | 1WK1 | 10328 | 2D7M | 11377 | 2EPP | 15740 | 2K36 | 16629 | 2KR7 | 17509 | 2LA7 | 18375 | 2LRJ | 19275 | 2M8V | 25156 | 2MTC | 4617 | 1DXW | 5676 | 1N8M | 6555 | 1YWS |
| 10037 | 1WJQ | 10329 | 2D7N | 11378 | 2EPQ | 15742 | 2K37 | 16633 | 2KRA | 17512 | 2LZN | 18378 | 4APD | 19282 | 2MTI | 25159 | 2MTF | 4622 | 1E52 | 5677 | 1K05 | 6557 | 1WZ4 |
| 10038 | 1WJP | 10330 | 2D7O | 11379 | 2EPR | 15743 | 2KP5 | 16641 | 2KRM | 17515 | 2LA8 | 18379 | 2LRK | 19283 | 2M96 | 25160 | 2MTG | 4628 | 1G9E | 5683 | 1NWB | 6561 | 1YTR |
| 10039 | 1WGQ | 10331 | 2D7P | 11380 | 2EPS | 15744 | 2L7B | 16642 | 2KRN | 17518 | 2LAA | 18387 | 2LRN | 19285 | 2M97 | 25168 | 2MU4 | 4636 | 1F2H | 5688 | 1N5P | 6564 | 2BO5 |
| 10040 | 1WGR | 10332 | 2D7Q | 11422 | 2RRK | 15746 | 2JPF | 16646 | 2KRR | 17521 | 2LAE | 18390 | 2LRQ | 19288 | 2M9A | 25188 | 2MTV | 4639 | 1FEX | 5691 | 1NY4 | 6573 | 1YXR |
| 10041 | 1WGS | 10333 | 2DI7 | 11423 | 2RRL | 15750 | 2K3D | 16647 | 2KRS | 17523 | 2LNC | 18393 | 2LRS | 19290 | 4BS2 | 25189 | 2MTW | 4640* | 1FJN | 5695 | 1NYN | 6574 | 1YVC |
| 10042 | 1WGV | 10334 | 2DI8 | 11424 | 2RRM | 15752 | 2JWH | 16648 | 2KRT | 17524 | 2LAH | 18394 | 2LRT | 19294 | 2M9H | 25191 | 2MTY | 4642 | 1FQQ | 5698 | 1NMR | 6581 | 2OI3 |
| 10043 | 1WGW | 10335 | 2DIA | 11426 | 2RRN | 15753 | 2JWG | 16649 | 2KRU | 17525 | 2LAI | 18398 | 2LRU | 19298 | 2M9K | 25194 | 2N2E | 4643 | 1FRY | 5701 | 1R9K | 6594 | 1Z2Q |
| 10044 | 1WH5 | 10336 | 1WWY | 11439 | 2RRS | 15762 | 2K3I | 16652 | 2KRX | 17530 | 2LAK | 18399 | 2LRW | 19299 | 2M9U | 25195 | 2MU0 | 4644 | 1FV5 | 5708 | 1O53 | 6596 | 1ZA8 |
| 10045 | 1WH7 | 10337 | 2E5K | 11450 | 2RS2 | 15765 | 2K3V | 16653 | 2L97 | 17531 | 2LAM | 18404 | 2LS0 | 19300 | 2M9L | 25200 | 2MU6 | 4645* | 1FWO | 5710 | 1OQK | 6597 | 2A24 |
| 10046 | 1WI0 | 11009 | 2RM4 | 11451 | 2RS4 | 15774 | 2K3M | 16667 | 2KSL | 17533 | 2LAQ | 18405 | 2LS1 | 19302 | 2M9M | 25203 | 2MU9 | 4648 | 1FZT | 5719 | 1S79 | 6598 | 1Z65 |
| 10047 | 2ECC | 11012 | 2RMN | 11452 | 2LIY | 15776 | 2K3R | 16672 | 2KSR | 17534 | 2LVL | 18408 | 2LS2 | 19311 | 2M9V | 25204 | 2MUA | 4651 | 1FDF | 5722 | 1OQP | 6609 | 1TTN |
| 10048 | 1WJI | 11017 | 2RN7 | 11456 | 2RS6 | 15777 | 2K3T | 16675 | 2KSV | 17537 | 2LAT | 18409 | 2LS3 | 19312 | 2M9W | 25206 | 2MUD | 4652 | 1EDX | 5729 | 1KFZ | 6613 | 1Z8R |
| 10049 | 1WJT | 11022 | 2RNG | 11457 | 2RS7 | 15778 | 2K3U | 16676 | 2KSW | 17547 | 2LB7 | 18411 | 2LS5 | 19314 | 2M9X | 25207 | 2MUE | 4654 | 1EDV | 5747 | 1NVO | 6615 | 1X37 |
| 10050 | 1WJU | 11024 | 2RNJ | 11458 | 2RS8 | 15790 | 2K48 | 16678 | 2KSY | 17551 | 2LBA | 18412 | 2LS7 | 19315 | 2M9Y | 25209 | 2MUF | 4656 | 1G26 | 5748 | 1RYU | 6624 | 2BYE |
| 10054 | 1WFQ | 11029 | 2RNN | 11468 | 2RSC | 15793 | 2K4B | 16679 | 2LFU | 17553 | 2LBB | 18415 | 2LS8 | 19319 | 2MA2 | 25210 | 4UZM | 4661 | 1C15 | 5750 | 1OSX | 6625 | 2AI6 |
| 10055 | 1WFS | 11030 | 2RNO | 11469 | 2RSD | 15801 | 2K4J | 16680 | 2KT0 | 17554 | 2LBC | 18416 | 2LWP | 19320 | 2MMY | 25211 | 2MUG | 4666 | 1DCZ | 5753 | 1NG7 | 6629 | 1ZDV |
| 10056 | 1WFY | 11032 | 2RO0 | 11471 | 2RSE | 15804 | 2K4M | 16681 | 2KT1 | 17557 | 2LBF | 18418 | 2LS9 | 19328 | 2MA5 | 25213 | 4UZW | 4668 | 1IX5 | 5758 | 1OVQ | 6631 | 1ZKH |
| 10057 | 1WFZ | 11033 | 2RNZ | 11472 | 2RSF | 15805 | 2K4N | 16686 | 2KT7 | 17561* | 2LBH | 18420 | 2LSA | 19329 | 2MA6 | 25214 | 4UZX | 4669 | 1CWX | 5765 | 1SU4 | 6635 | 2BYF |
| 10058 | 1WIK | 11034 | 2RO5 | 11473 | 2RSG | 15807 | 2K4Q | 16688 | 2KT8 | 17585 | 2LC0 | 18425 | 2LUC | 19331 | 2MA7 | 25217 | 2MUK | 4677 | 1EXK | 5766 | 1NZP | 6636* | 1X5V |
| 10059 | 1WIN | 11038 | 2ROH | 11477 | 2RUX | 15810 | 2K4V | 16691 | 2KT9 | 17586 | 2LC1 | 18429 | 2LSE | 19333 | 2MA9 | 25221 | 2MUM | 4679 | 1FM1 | 5767 | 1ORL | 6644 | 2MUI |
| 10060 | 1WJK | 11040 | 2ROL | 11478 | 2RUY | 15811 | 2K4X | 16709 | 2KTN | 17588 | 2LC2 | 18435 | 2LSL | 19340 | 2MAB | 25223 | 2MUN | 4688 | 1ILY | 5771 | 1E9T | 6645 | 2ITH |
| 10063 | 1UE9 | 11041 | 2ROP | 11479 | 2RUZ | 15812 | 2K4Y | 16711 | 2KTS | 17594 | 2LC3 | 18437 | 2LSM | 19342 | 2MAE | 25235 | 2MUY | 4700 | 1CF4 | 5779 | 1NNV | 6649 | 1X60 |
| 10064 | 1UEW | 11042 | 2KHE | 11480 | 2RV0 | 15816 | 2K4Z | 16721 | 2KU3 | 17595 | 2LCQ | 18438 | 2LSO | 19354 | 2MAH | 25238 | 2MV1 | 4701 | 1D9S | 5786 | 1OP4 | 6654 | 1ZJQ |
| 10065 | 1UFN | 11044* | 2ROO | 11481 | 2RV1 | 15819 | 2K50 | 16731 | 2KUC | 17596 | 2LSH | 18441 | 2LSR | 19356* | 4BWH | 25240 | 2MV2 | 4706 | 1CX1 | 5787 | 1Z5F | 6655 | 1ZU1 |
| 10066 | 1UFX | 11054 | 2RPS | 11482 | 2RV2 | 15821 | 2K52 | 16732 | 2KUD | 17598 | 2LC4 | 18442 | 2LSS | 19365 | 2MAL | 25241 | 2MV3 | 4716 | 1N4C | 5796 | 1PA4 | 6682 | 1ZR9 |
| 10067 | 1UHT | 11060 | 2RPB | 11483 | 2RV3 | 15825 | 2K57 | 16737 | 2KXI | 17603 | 2LC9 | 18443 | 2LST | 19366 | 2MAO | 25242 | 4V10 | 4717 | 1F7W | 5798 | 1NY8 | 6690 | 2BN5 |
| 10068 | 1UJO | 11061 | 2RPZ | 11485 | 2RV5 | 15828 | 2K5C | 16745 | 2KUS | 17606 | 2LCC | 18459 | 4AR0 | 19368 | 4BXU | 25254 | 2MVA | 4721 | 1D8J | 5807 | 1VD0 | 6696 | 2BUG |
| 10069 | 1UJS | 11065 | 2RQ1 | 11486 | 2RUV | 15833 | 2K5E | 16746 | 2KUT | 17607 | 2MAM | 18463 | 2LT8 | 19372 | 2MAR | 25265 | 2MVF | 4735 | 1JYT | 5817 | 1PB5 | 6714 | 2A1C |
| 10070 | 1WEZ | 11073 | 2RQF | 11491 | 2RSM | 15834 | 2K5F | 16767 | 2KV3 | 17609 | 2LCE | 18464 | 2LT9 | 19374* | 2MAU | 25266 | 2MVG | 4740 | 1GH9 | 5820 | 1PJZ | 6715 | 1ZZA |
| 10071 | 1WF1 | 11077 | 2RQL | 11496 | 2RSN | 15835 | 2K5G | 16774 | 2KVC | 17610 | 2N46 | 18465 | 2LTA | 19379 | 2MAW | 25275 | 2MVM | 4743 | 1EHJ | 5824 | 1Q6A | 6716 | 1Z8S |
| 10072 | 1WF2 | 11083 | 2ELL | 11497 | 2RSO | 15836 | 2K5H | 16778 | 2KVM | 17611 | 2N4B | 18468 | 2LTE | 19380 | 2MAX | 25277 | 2MVO | 4751 | 1IVM | 5833 | 1R3B | 6717 | 1ZG2 |
| 10073 | 1WFJ | 11087 | 2YS3 | 11502 | 2RSQ | 15837 | 2K5I | 16779 | 2LXD | 17612 | 2LCH | 18474 | 2M1H | 19382 | 2MB0 | 25281 | 2N2M | 4752 | 1J9I | 5836 | 1POQ | 6720 | 1ZLC |
| 10074 | 1WFM | 11088 | 2YS1 | 11504 | 2RT5 | 15839 | 2K5J | 16782 | 2KVO | 17614 | 2LCK | 18475 | 2LTF | 19390 | 2N2J | 25283 | 2MVT | 4754 | 1J5L | 5842 | 1Q48 | 6721 | 1ZR7 |
| 10075 | 1WFN | 11090 | 2YS4 | 11507 | 2RSX | 15841 | 2K5L | 16789 | 2KVR | 17615 | 2LCL | 18484 | 2LTJ | 19391 | 2MB7 | 25288 | 2MVW | 4757 | 1DV0 | 5843 | 1Q53 | 6726 | 1ZXF |
| 10076 | 1WFO | 11091 | 2E6J | 11508 | 2RSY | 15843 | 2N49 | 16790 | 2X8N | 17617 | 2LCM | 18492 | 2LTP | 19392 | 2MB9 | 25293 | 2MW0 | 4760 | 1C49 | 5844 | 2M6Q | 6727 | 2AJE |
| 10077 | 1WI8 | 11092 | 2EFI | 11523 | 2RT3 | 15844 | 2K5P | 16791 | 2KVT | 17622 | 2YH0 | 18497 | 2LTU | 19396 | 2MBD | 25298 | 2MW4 | 4771 | 1S62 | 5845 | 1R57 | 6729 | 2E2F |
| 10079 | 1V9V | 11093 | 2E6I | 11525 | 2RT6 | 15847 | 2K5R | 16794 | 2KVS | 17625 | 2YHH | 18504 | 2LU1 | 19397 | 2MBE | 25302 | 2MW7 | 4779 | 1PLO | 5847 | 1PV0 | 6731 | 2BW2 |
| 10080 | 1V9W | 11094 | 2YS5 | 11529 | 2RT9 | 15849 | 2K5V | 16795 | 2KVV | 17628 | 2LCR | 18511 | 2LU7 | 19398 | 2MBF | 25307 | 2MW8 | 4791 | 1JR6 | 5848 | 1UGL | 6737* | 2A2B |
| 10081 | 1VAE | 11095 | 2YT2 | 11530 | 2RTS | 15850 | 2K5W | 16797 | 2KVH | 17629 | 2LCS | 18514 | 2LUA | 19403 | 2MBK | 25308 | 2MWC | 4792 | 2AN7 | 5849 | 1UFM | 6738 | 2A4H |
| 10082 | 1WEY | 11096 | 2ED7 | 11531 | 2RTT | 15851 | 2K60 | 16801 | 2KVZ | 17632 | 2LCT | 18521 | 2LUH | 19413 | 2MBS | 25312 | 4D4W | 4797 | 1KD6 | 5850 | 1P8A | 6739 | 2A2P |
| 10083 | 1WFG | 11097 | 2EDD | 11534 | 2RTX | 15855 | 2KG4 | 16802 | 2KW0 | 17633 | 2LCU | 18522 | 2LUI | 19415 | 2MBV | 25324 | 2MWH | 4798 | 1FPW | 5851 | 1P7A | 6740 | 2A05 |
| 10084 | 1WFI | 11098 | 2EDE | 11535* | 2RTY | 15863 | 2K6A | 16808 | 2KW6 | 17635 | 2LCW | 18526 | 2LUL | 19416 | 2MBY | 25326 | 2MWI | 4814 | 1LS4 | 5861 | 1RFL | 6741 | 2JOU |
| 10085 | 1WG5 | 11099 | 2EDB | 11536* | 2RTZ | 15864 | 2K6B | 16812 | 2KAE | 17636 | 2LV3 | 18533 | 2LUO | 19425 | 2MC2 | 25350 | 2MWQ | 4825 | 1M58 | 5865* | 1PVZ | 6743 | 2ADN |
| 10086 | 1WGY | 11100 | 2DML | 11537 | 2RU0 | 15870 | 2K6I | 16817 | 2KSG | 17645 | 2LD3 | 18539 | 2LUS | 19427 | 2MC4 | 25352 | 2MWR | 4833 | 1G6E | 5866 | 1PPX | 6746 | 2AKK |
| 10088 | 1WHA | 11101 | 2DJ1 | 11538 | 2MDB | 15880 | 2K6S | 16822 | 2KGK | 17646 | 2LD4 | 18540 | 2LUT | 19432 | 2MC9 | 25363* | 2MWT | 4837 | 1E8E | 5868 | 1PVE | 6748 | 2AFF |
| 10089 | 1WJ1 | 11102 | 2DMM | 11540 | 2RU1 | 15893 | 2K6V | 16824 | 2K42 | 17651 | 2LD6 | 18541 | 2LUU | 19436 | 2MCD | 25372 | 4D7X | 4841 | 1E91 | 5869 | 1PQR | 6749 | 2CZY |
| 10090 | 1WJJ | 11103 | 2ED8 | 11544 | 2RU4 | 15900 | 2K72 | 16831 | 2KNG | 17661 | 2LDE | 18542 | 2LUV | 19437* | 2MCE | 25376 | 2MWX | 4880 | 1H67 | 5873 | 1P6R | 6751 | 2AFE |
| 10091 | 1WJN | 11104 | 2DJ2 | 11549 | 2RU8 | 15902 | 2K75 | 16832 | 2KTL | 17662 | 2LDF | 18546 | 2LUY | 19438 | 2MCF | 25380 | 2MX0 | 4890 | 1G25 | 5874 | 1VAZ | 6752 | 1Z87 |
| 10092 | 1WH4 | 11105 | 2DJ0 | 11555 | 2RU9 | 15913 | 2K78 | 16833 | 2LOY | 17667 | 2LL4 | 18547 | 2LUZ | 19449 | 2MCQ | 25381 | 2MX1 | 4891 | 1G6M | 5876 | 1V92 | 6753 | 2ADZ |
| 10093 | 1WH6 | 11106 | 2DMK | 11569 | 2RUH | 15916 | 2K7I | 16839 | 2KUM | 17668 | 2LDI | 18548 | 2M5H | 19450 | 2MCR | 25395 | 2MX7 | 4893 | 1KVZ | 5878 | 1SSF | 6755 | 2ABY |
| 10094 | 1WH8 | 11107 | 2DIZ | 11570 | 2RUI | 15922 | 2K7N | 16840 | 2L9C | 17670 | 2LDK | 18551 | 2LV2 | 19453 | 2MCT | 25396 | 2MXB | 4894 | 1EKZ | 5880 | 1UHU | 6757 | 2AJJ |
| 10095 | 1WH9 | 11108 | 2DIY | 11583 | 2RUP | 15925 | 2K7Q | 16842 | 2LDO | 17673 | 2LDM | 18553 | 2LV4 | 19460 | 2MD0 | 25397 | 2MX8 | 4895 | 1G6P | 5881 | 1Q2J | 6759 | 2H3K |
| 10097 | 1J0F | 11109 | 2DJ3 | 11590 | 2RV8 | 15926 | 2K7R | 16853 | 2KLU | 17679 | 2MKN | 18555 | 2LV5 | 19464 | 4C26 | 25400 | 2N93 | 4900 | 1HEH | 5882 | 2OII | 6760 | 2NPU |
| 10098 | 1R79 | 11110 | 2CUM | 11591 | 2RV9 | 15931 | 2K7V | 16856 | 2KWL | 17680 | 2LDR | 18557 | 2LV7 | 19468 | 2MD1 | 25402 | 2MXC | 4902 | 1RJA | 5885 | 1PZR | 6761 | 2AL3 |
| 10099 | 1UEM | 11111 | 1X5K | 11592 | 2RVA | 15932 | 2K7Z | 16857 | 2KB9 | 17683 | 2LDU | 18559 | 2LV9 | 19479 | 2MD9 | 25404 | 2MXD | 4906 | 1HF9 | 5891 | 1XSX | 6762 | 1Z4H |
| 10100 | 1UEP | 11112 | 1X5I | 11595 | 2RVC | 15939 | 2K86 | 16860 | 2LML | 17685 | 2LWX | 18560 | 2LVA | 19484 | 2MDF | 25419 | 2MXM | 4907 | 1FJP | 5892 | 2A2Y | 6766 | 2AKL |
| 10101 | 1UG7 | 11113 | 1X5E | 11601 | 2RVJ | 15941 | 2K8D | 16864 | 2MA0 | 17686 | 2LDY | 18563 | 2LVC | 19486 | 2MDG | 25423 | 2MXP | 4910 | 1HDL | 5901 | 1PXE | 6776 | 2BZT |
| 10102 | 1UJT | 11115 | 2RR4 | 15000 | 2JM0 | 15943 | 2K8E | 16868 | 2KWP | 17687 | 2LE0 | 18566 | 2M5S | 19489 | 2MDK | 25424 | 2MXQ | 4915 | 1G9L | 5902 | 1RI0 | 6780 | 2OUT |
| 10103 | 1UJU | 11116 | 1X5D | 15007 | 2NPR | 15946 | 2K76 | 16871 | 2JWE | 17689 | 2LE2 | 18568 | 2LVG | 19498 | 2MDP | 25433 | 2MXV | 4917 | 1BV2 | 5905 | 1SAP | 6781 | 2AQF |
| 10104 | 1UJX | 11117 | 1X5A | 15016 | 2JMF | 15948 | 2KIZ | 16881 | 2KF2 | 17690 | 2LE3 | 18570 | 2LVH | 19501 | 2MDQ | 25434 | 2MXW | 4919 | 1G7E | 5907 | 1RW2 | 6797 | 2PXG |
| 10105 | 1V31 | 11118 | 1X5J | 15021 | 2I7U | 15950 | 2K8I | 16886 | 2KWT | 17691 | 2LE4 | 18581 | 2LVN | 19502 | 2MDR | 25435 | 2MXX | 4920 | 2M66 | 5911 | 1Q2I | 6803 | 2K1X |
| 10106 | 1V32 | 11119 | 1X5F | 15023 | 2JML | 15956 | 2K3G | 16892 | 2KWZ | 17694 | 2LS6 | 18586 | 2LVR | 19503 | 2MDU | 25436 | 2MXY | 4921 | 1G92 | 5920 | 1UMQ | 6805 | 2B89 |
| 10107 | 1V5K | 11120 | 1X5H | 15025 | 2NVJ | 15961 | 2MPO | 16897 | 2KX2 | 17699 | 2LE7 | 18587 | 2M0A | 19504 | 2MDV | 25437 | 2MXZ | 4923 | 1G9P | 5922* | 1UJL | 6809 | 2FIN |
| 10108 | 1V5N | 11121 | 1X5G | 15032 | 2JMR | 15966 | 2K73 | 16899 | 2KX3 | 17703 | 2LTR | 18589 | 2LVS | 19505 | 2MDW | 25439 | 2MY1 | 4924 | 1HP2 | 5933 | 1X9X | 6810 | 2AYX |
| 10109 | 1V5Q | 11122 | 2DBA | 15038 | 2OFN | 15981 | 2K8Y | 16901 | 2K6T | 17713 | 4BF8 | 18590 | 4B19 | 19523 | 2MEK | 25449 | 2MYF | 4927 | 1N02 | 5935 | 1BIF | 6811 | 2AJ0 |
| 10110 | 1V5R | 11123 | 2YUS | 15045 | 2JMM | 15983 | 2K9E | 16905 | 2LPM | 17717 | 2LEM | 18591 | 2LVW | 19525 | 4C7Q | 25451 | 2MYG | 4929 | 1XDX | 5939 | 2JP2 | 6812 | 2FQH |
| 10111 | 1V63 | 11144 | 2YUA | 15055 | 2JMP | 15987 | 2K9I | 16907 | 2KXA | 17719 | 2LEO | 18592 | 2LVX | 19526 | 2MEM | 25452 | 2MYH | 4935 | 1KVV | 5940 | 2ARW | 6816 | 2AHQ |
| 10112 | 1V86 | 11150 | 2YS0 | 15058 | 2JMS | 15988 | 2K9K | 16909 | 2KXC | 17720 | 2LEP | 18600 | 4B2U | 19533 | 2MEW | 25454 | 2MYJ | 4938 | 1I17 | 5944 | 1Q68 | 6829 | 2CZN |
| 10113 | 1V5M | 11151 | 2YUU | 15059 | 2O13 | 15989 | 2K9N | 16911 | 2LKL | 17723 | 2LEQ | 18602 | 2LW3 | 19535 | 2MF3 | 25456* | 2MYL | 4939 | 1FU9 | 5946 | 2FVN | 6835 | 2AMN |
| 10114 | 1V5U | 11152 | 2YRZ | 15074 | 2JMO | 15992 | 2K9O | 16912 | 2M4K | 17727 | 2LES | 18605 | 2LW4 | 19536 | 2MF4 | 25459 | 2MYV | 4941 | 1JJR | 5947 | 1RXL | 6841 | 2B1U |
| 10115 | 1V88 | 11154 | 2ECZ | 15079 | 2JN0 | 15996 | 2WCY | 16922 | 2KSF | 17729 | 2LEV | 18606 | 2LW5 | 19538 | 2MF7 | 25460 | 2MYW | 4945 | 1HS7 | 5950 | 2BN8 | 6843 | 2B1W |
| 10118 | 2EXD | 11155 | 2ED0 | 15084 | 2JN3 | 15999 | 2K9Q | 16923 | 2KXQ | 17734 | 2LEZ | 18607 | 2LW6 | 19539 | 4CA3 | 25461 | 2MYX | 4946 | 1HZK | 5956 | 1R05 | 6844 | 2AYM |
| 10119 | 1IUR | 11156 | 2YUN | 15085 | 2JN4 | 16001 | 2K9S | 16927 | 2KXY | 17736 | 2LF2 | 18612 | 2LW7 | 19540 | 2MF8 | 25464 | 2MYY | 4954 | 1HZE | 5961 | 2FYJ | 6845 | 2AQC |
| 10121 | 1J26 | 11158 | 2YUL | 15086 | 2JN6 | 16007 | 2KA0 | 16929 | 2KXV | 17737 | 2LF3 | 18613 | 2LW8 | 19542 | 2MFA | 25468 | 2MZ0 | 4957 | 1I1S | 5963 | 2APN | 6846 | 2AQA |
| 10122 | 1J3T | 11159 | 2YUR | 15088 | 2JN7 | 16019 | 2KA5 | 16935 | 2KY5 | 17739 | 2LF6 | 18614 | 2LW9 | 19551 | 2MPH | 25476 | 2MZ8 | 4972 | 1K1C | 5964 | 1UNC | 6849* | 2B68 |
| 10123 | 1N27 | 11160 | 2YUK | 15089 | 2JN8 | 16024 | 2KAJ | 16936 | 2KY8 | 17745 | 2LSN | 18622 | 2LWD | 19552 | 2MFJ | 25482 | 2MZB | 4977 | 1N89 | 5966 | 1UND | 6860 | 2GW6 |
| 10124 | 1UJV | 11161 | 2ECW | 15090 | 2MA8 | 16025 | 2KAK | 16942 | 2KY9 | 17747 | 2LFC | 18624 | 2LWF | 19553 | 2MFK | 25496 | 2MZQ | 4979 | 1HA8 | 5970 | 1OVY | 6865 | 2BBX |
| 10125 | 1WHN | 11162 | 2ECT | 15098 | 2CKA | 16028* | 2KAM | 16945 | 2KYC | 17750 | 2LFE | 18636 | 2N0M | 19555 | 2MFM | 25497 | 2MZR | 4984 | 1KOS | 5971 | 1Z66 | 6867 | 2ARI |
| 10126 | 1WHR | 11163 | 2ECV | 15103 | 2NX6 | 16029 | 2N4F | 16947 | 2KSD | 17752 | 2LFG | 18641 | 2LWQ | 19558 | 2MYI | 25498 | 2MZS | 4988 | 1I25 | 5972 | 1R48 | 6868 | 2ASY |
| 10127 | 1WHU | 11164 | 2ECY | 15104 | 2NX7 | 16030 | 2KAT | 16954 | 2KYG | 17753 | 2LFH | 18644 | 2LWT | 19568 | 2MFR | 25499 | 2MZT | 4989 | 1HY8 | 5977 | 1R73 | 6869 | 2AYA |
| 10128 | 1VA9 | 11166 | 2EOL | 15105 | 2IKD | 16032 | 2KAV | 16956 | 2ML3 | 17754 | 2LFI | 18645 | 2LWU | 19570* | 2MFS | 25504 | 2MZW | 4991 | 1ILO | 5978 | 1R7C | 6872 | 2B38 |
| 10129 | 1WF5 | 11170 | 2EP3 | 15106 | 2IKE | 16037 | 2KB2 | 16957 | 2KYH | 17763* | 2LFK | 18651 | 2MME | 19573 | 2MFV | 25508 | 2MZY | 4995 | 1IHQ | 5981 | 1YHO | 6876 | 2FO8 |
| 10130 | 1WF9 | 11178 | 1UGV | 15108 | 2IMU | 16038 | 2KB3 | 16960 | 2WH9 | 17768 | 2LFP | 18653 | 2LWY | 19579 | 2MFZ | 25509 | 2MZZ | 4996 | 1IIO | 5985 | 1UL2 | 6882 | 2ERS |
| 10131 | 1V5P | 11179 | 1WI7 | 15109 | 2JNG | 16058 | 2KC0 | 16967 | 2KYL | 17772 | 3ZTG | 18655 | 2LX0 | 19581 | 2MG1 | 25510 | 2N00 | 5004 | 1IK0 | 5989 | 1RL5 | 6884 | 2FHM |
| 10136 | 1UFF | 11180 | 1WIE | 15111 | 2JNH | 16061 | 2KC5 | 16969 | 2XA6 | 17775 | 2LFR | 18657 | 2LX2 | 19582 | 2MG2 | 25527 | 2N0K | 5012 | 1J7M | 5991 | 1R36 | 6885 | 2CKN |
| 10138 | 2DOG | 11182 | 2CSS | 15112 | 2K4W | 16063 | 2KBX | 16970 | 2KYM | 17783 | 2LFV | 18658 | 2LX4 | 19585 | 2MG4 | 25529 | 2N0O | 5018 | 1ICH | 5998 | 2F1E | 6892 | 2ESY |
| 10141 | 2E9G | 11183 | 2CSP | 15124 | 2JNR | 16065 | 2KC8 | 16971 | 2L8B | 17784 | 2LFW | 18659 | 2LX5 | 19600 | 2MGQ | 25530 | 2N0P | 5022 | 1I4V | 6001 | 1XWE | 6893 | 2BAF |
| 10142 | 2E9I | 11184 | 2CSQ | 15125 | 2JNS | 16068 | 2KCA | 16977 | 2L8K | 17797 | 2LG5 | 18661 | 2LX6 | 19606 | 2MGW | 25539 | 2N0X | 5027 | 1J8K | 6002 | 1RG6 | 6894 | 2B3A |
| 10143 | 2E9J | 11185 | 2CSI | 15126 | 2OQ3 | 16072 | 2KCD | 16981 | 2KYS | 17809 | 2LGH | 18662 | 2LX7 | 19607 | 2MGX | 25543 | 2N11 | 5030 | 1TUJ | 6004 | 1Q8G | 6895 | 2ERR |
| 10146 | 2EE6 | 11186 | 2D92 | 15128 | 2JNU | 16073 | 2KCG | 16983 | 2KYU | 17811 | 2LGJ | 18668 | 2LX9 | 19608 | 2MGY | 25552 | 2N19 | 5031 | 1M2E | 6005 | 1RGO | 6910 | 2FE0 |
| 10147 | 2EE7 | 11187 | 2DAZ | 15133 | 2JNY | 16074 | 2KCH | 16988 | 2KYW | 17816 | 2LGN | 18672 | 2LXE | 19610 | 2MH0 | 25555 | 2N1C | 5033 | 1I2U | 6007 | 1RJH | 6914 | 2ARF |
| 10148 | 2EE8 | 11188 | 2DB5 | 15134 | 2JNZ | 16082 | 2KCJ | 16989 | 2KYX | 17818 | 2LGO | 18673 | 2LXF | 19613 | 2MH2 | 25564 | 2N1K | 5036 | 1I11 | 6010 | 1RHX | 6915* | 2LA1 |
| 10149 | 2E9K | 11189 | 2DLU | 15139 | 2JO6 | 16083 | 2KCK | 16995 | 2KZ0 | 17824 | 2LGV | 18677 | 2LXH | 19614 | 2MH3 | 25565 | 2N1L | 5041 | 1IN1 | 6011 | 1RJJ | 6916 | 2EWL |
| 10150 | 2ELK | 11190 | 2DMZ | 15140* | 2EEM | 16084 | 2KCL | 16996 | 2KZ3 | 17825 | 2LGW | 18678 | 2LXI | 19618 | 2MI2 | 25568 | 2N1N | 5043 | 1H9F | 6012 | 1RL1 | 6919 | 2CH0 |
| 10151 | 2EN6 | 11191 | 2DM8 | 15152 | 2OT2 | 16085 | 2ML8 | 16998 | 2KZ5 | 17827 | 2LGX | 18684 | 2LXO | 19619 | 2MH5 | 25570 | 2N1P | 5044 | 1IE5 | 6015 | 2AVG | 6920 | 2FFW |
| 10152 | 2EN7 | 11192 | 2DLT | 15154 | 2JOA | 16089 | 2KCO | 16999 | 2KZ6 | 17833 | 2N4I | 18692 | 2LXR | 19621 | 2MHF | 25575 | 2N1U | 5047 | 1JBI | 6020 | 1RKK | 6922 | 2FE9 |
| 10153 | 2EOV | 11193 | 2EGA | 15158 | 2JO7 | 16091 | 2KCP | 17000 | 2KZ9 | 17835 | 2LH9 | 18698 | 2LXU | 19622 | 2N0S | 25585 | 2N24 | 5049 | 1N6U | 6024 | 1ERO | 6923 | 2MF9 |
| 10154 | 2EOY | 11194 | 2EGC | 15160 | 2I5O | 16094 | 2KCR | 17001 | 2KZA | 17842 | 2LHF | 18700 | 2LXW | 19623 | 2MH8 | 25593 | 2N2A | 5050* | 1JC6 | 6028 | 1RQ6 | 6924* | 2AXK |
| 10155 | 2EP2 | 11195 | 2DLS | 15166 | 2JOD | 16096 | 2KCT | 17002 | 2KZC | 17855 | 2LHJ | 18702 | 4B8T | 19625 | 2MHC | 25597 | 2N2F | 5051 | 1JCU | 6029 | 1SVJ | 6925 | 2C06 |
| 10156 | 2YTS | 11196 | 2EGE | 15167 | 2JOE | 16097 | 2KCU | 17005 | 2KZH | 17858 | 2LHN | 18707 | 2LY3 | 19626 | 2MJF | 25605 | 2N2Q | 5053 | 1IFW | 6032 | 1WM4 | 6927 | 2G5M |
| 10157 | 2EOS | 11197 | 2EHR | 15174 | 2P4L | 16098 | 2KCW | 17006 | 2KZB | 17865 | 2LHT | 18711 | 4BA8 | 19627 | 2MHD | 25611 | 2N2T | 5055 | 1IMU | 6034 | 1QTS | 6929 | 2FGX |
| 10158 | 2EOU | 11198 | 1UM7 | 15189 | 2JOP | 16099 | 2KCX | 17007 | 2KZK | 17867 | 2LHU | 18712 | 2LY7 | 19628 | 2MHE | 25612 | 2N2U | 5060 | 1JDQ | 6037 | 1RJI | 6934 | 2FEK |
| 10159 | 2EE9 | 11200 | 1VA8 | 15195* | 2EFZ | 16100 | 2KCZ | 17008 | 2KZN | 17880 | 2LI6 | 18713 | 2LY8 | 19632 | 2MHG | 25627 | 2N2Y | 5065 | 1IBI | 6038 | 1RJT | 6938 | 2V1N |
| 10160 | 2EEA | 11202 | 1WF8 | 15203 | 2JOV | 16101 | 2KD0 | 17011 | 2KZQ | 17883 | 2LI8 | 18714 | 2LY9 | 19633 | 2MHH | 25630 | 2N2Z | 5066 | 1JI9 | 6043 | 2P80 | 6943 | 2JNT |
| 10161 | 2EEB | 11203 | 1X5M | 15208 | 2JOX | 16102 | 2KD1 | 17014 | 2M1B | 17886 | 2M5R | 18717 | 2LYC | 19635 | 2MHJ | 25634 | 2N35 | 5070 | 1JH3 | 6045 | 1S04 | 6945 | 2FS1 |
| 10162 | 2EEC | 11204 | 1X5N | 15210 | 2JOY | 16103 | 2KD2 | 17015* | 2K38 | 17888 | 2LIC | 18720 | 2LYD | 19637 | 2MHL | 25642 | 2N3D | 5072 | 1JBJ | 6046 | 1S6N | 6951* | 2FQC |
| 10163 | 2EED | 11205 | 2COT | 15211 | 2JOZ | 16109 | 2KDB | 17016 | 2KZ7 | 17889 | 2LID | 18725 | 2LYH | 19642 | 2MHN | 25645 | 2N3J | 5074 | 1JEI | 6052 | 1SGO | 6952 | 2FQA |
| 10165 | 2EEF | 11206 | 1X5R | 15229 | 2NXU | 16110 | 2KDD | 17017 | 2KZR | 17891 | 2LIF | 18726 | 2LYI | 19643 | 2MHO | 25656 | 2N3S | 5075 | 1JFN | 6058 | 1RZW | 6955 | 2F09 |
| 10166 | 2EM1 | 11207 | 1X5Q | 15240 | 2JPD | 16111 | 2KPY | 17020 | 2KZV | 17899 | 2MU3 | 18728 | 2LYV | 19646 | 2MHP | 25657 | 2N3T | 5077 | 1JJG | 6059 | 1RFH | 6957 | 2FI2 |
| 10167 | 2EM2 | 11208 | 1X5L | 15242 | 2JPE | 16118 | 2KDN | 17021 | 2KZW | 17900 | 2LIO | 18729 | 4B2V | 19654 | 2MHS | 25662 | 2N3Z | 5078 | 1K8O | 6063 | 1RIM | 6963 | 2DDJ |
| 10168 | 2EM3 | 11209 | 1X43 | 15246 | 2JPI | 16122 | 2KDR | 17023 | 2KZY | 17908 | 2LIX | 18733 | 2LYY | 19658 | 2MHW | 25670 | 2N4K | 5082* | 1JLZ | 6064 | 1RIK | 6964 | 2FVT |
| 10169 | 2EN8 | 11210 | 1X45 | 15247 | 2V9H | 16126 | 2KDX | 17025 | 2L01 | 17911 | 2LIZ | 18735 | 2LZ1 | 19660 | 2MHY | 25675 | 2N4P | 5086 | 1AYG | 6072 | 1V66 | 6969 | 2FXP |
| 10170 | 2EN9 | 11211 | 1X44 | 15252 | 2JPN | 16127 | 2KDP | 17026 | 2L02 | 17912 | 2LJ0 | 18736 | 2LZ5 | 19663 | 2MI1 | 25684 | 5A4H | 5092* | 1JO6 | 6079 | 1TI3 | 6971 | 2DJK |
| 10171 | 2ENA | 11212 | 2DAV | 15258 | 2JPQ | 16129 | 2KE4 | 17028 | 2L04 | 17918 | 2LJ4 | 18749 | 2LZE | 19666 | 2MI5 | 25688 | 2N50 | 5094 | 1K19 | 6081 | 2JNF | 6976 | 2FJ6 |
| 10173 | 2ENE | 11213 | 2D8H | 15259* | 2JPJ | 16135 | 2KMT | 17030 | 2L05 | 17923 | 2LJ7 | 18753 | 2LZF | 19667 | 2MI6 | 25691 | 2N52 | 5097* | 1IJC | 6082* | 1S6D | 6980 | 2G2B |
| 10175 | 2ENH | 11215 | 2D8I | 15261* | 2JPK | 16137 | 2KE7 | 17031 | 2L06 | 17926 | 2LJ9 | 18763 | 2LZL | 19674 | 2MID | 25699 | 2N58 | 5100 | 1J2O | 6084 | 1SQ8 | 6981 | 2G0U |
| 10176 | 2YTM | 11216 | 2D90 | 15268 | 2JQ3 | 16141 | 2KEA | 17033 | 2L08 | 17927 | 2LJA | 18764 | 4BD3 | 19679 | 2MIH | 25706 | 2N5D | 5104 | 1JRM | 6085 | 1S6W | 6982 | 2G1E |
| 10178 | 2YTT | 11217 | 2DL4 | 15269 | 2JQ4 | 16153 | 2KEL | 17035 | 2L09 | 17928 | 2LJB | 18766 | 2LZO | 19681 | 2MII | 25712 | 2N5F | 5106 | 1RYJ | 6088 | 1RIJ | 6989 | 2G1D |
| 10179 | 2EM4 | 11218 | 2EEL | 15275 | 2JQE | 16155 | 2KEO | 17038 | 2L0C | 17935 | 2LXN | 18768 | 2LZP | 19682 | 2MIQ | 25716 | 2N5H | 5112 | 1JLP | 6090 | 2GT3 | 6991 | 2CEH |
| 10180 | 2EM7 | 11219 | 2DL3 | 15279 | 2JQ6 | 16156 | 2KES | 17039 | 2L0D | 17936 | 2LJH | 18769 | 2LZQ | 19683 | 2MIJ | 25718 | 2N5J | 5113* | 1JLO | 6102 | 1RWU | 6997 | 2G46 |
| 10181 | 2EM9 | 11221 | 2E6Q | 15281 | 2JQN | 16161 | 2KEY | 17040 | 2L0E | 17942 | 2M3M | 18776 | 2MPU | 19684 | 2MIO | 25719 | 2N5K | 5115 | 1JI8 | 6104 | 1S6I | 7000 | 2A7Y |
| 10182 | 2EME | 11223 | 2DL9 | 15288 | 2JQO | 16168 | 2JO1 | 17045 | 2LPE | 17956 | 2LJS | 18778 | 2LZU | 19687 | 2MIM | 25720 | 2N5L | 5129 | 1IQS | 6108 | 1RRZ | 7001 | 2G57 |
| 10183 | 2EMF | 11224 | 2EQI | 15290 | 2JQQ | 16173 | 2KFB | 17050 | 2XK0 | 17962 | 2LJU | 18782 | 2YMJ | 19688 | 2MNJ | 25732 | 2N5U | 5141 | 1T4Z | 6109* | 1S8K | 7007 | 2G0Q |
| 10184 | 2EMG | 11225 | 2DL8 | 15295 | 2JQV | 16176 | 2KFD | 17066 | 2L0W | 17971 | 2LK2 | 18784 | 2LZY | 19689 | 2MZV | 25740 | 2N5X | 5145 | 1LG4 | 6114 | 1OQA | 7008 | 2FWU |
| 10185 | 2YTF | 11227 | 2E7H | 15299* | 2Z3S | 16178* | 2KFE | 17070 | 2L10 | 17973 | 2LT5 | 18794 | 2M05 | 19694 | 2MIU | 25744 | 2N5Z | 5147 | 1JJD | 6120 | 1T17 | 7014 | 2GDT |
| 10186 | 2YTG | 11228 | 2YSQ | 15302 | 2JQW | 16186 | 2KFP | 17071 | 2L11 | 17987 | 2LO7 | 18802 | 2M09 | 19697 | 2MIX | 25748 | 2N62 | 5148 | 1JQ4 | 6121 | 2DHS | 7033 | 3THK |
| 10189 | 2EOM | 11229 | 2E6P | 15306 | 2JQZ | 16187* | 2KFQ | 17089 | 2KXD | 17988 | 2LKB | 18805 | 2M0C | 19699 | 2MIZ | 25758 | 2N6B | 5159 | 1XKE | 6122 | 1SLJ | 7051 | 2DJC |
| 10191 | 2EOP | 11230 | 2E7B | 15312 | 2JR0 | 16188 | 2KFS | 17090 | 2L1N | 17989 | 2LW1 | 18811 | 2M0M | 19700* | 2MJ0 | 25762 | 2N6F | 5162 | 1MP1 | 6123 | 1HEV | 7054 | 2GM2 |
| 10192 | 2EOQ | 11231 | 2YRL | 15314 | 2JR1 | 16189 | 2KFV | 17091 | 2L1O | 17990 | 2LKJ | 18812 | 2M0N | 19702* | 2MJ2 | 25763 | 2N6G | 5165 | 1JW3 | 6127 | 1SE7 | 7056 | 2GMO |
| 10193 | 2YUM | 11232 | 2E7M | 15317 | 2JR2 | 16191 | 2KLE | 17092 | 2L1P | 17993 | 2LQ4 | 18813 | 2M0O | 19703 | 2MJ3 | 25766 | 2N6J | 5166 | 1JW2 | 6128 | 1SE9 | 7063 | 2G7J |
| 10194 | 2YUO | 11234 | 2YSL | 15318 | 2JR3 | 16195 | 2LG1 | 17104 | 2L1S | 18000 | 2LKM | 18816 | 2M0P | 19707 | 2MJ6 | 25768 | 2N6L | 5172 | 1K3G | 6134 | 1SA8 | 7064 | 2GJ0 |
| 10195 | 2YUP | 11235 | 2E7C | 15320 | 2JR5 | 16211 | 2KGJ | 17105 | 2L1T | 18001 | 2LKN | 18817 | 2M0Q | 19709 | 2MJ7 | 25786 | 2N6Y | 5173* | 1K36 | 6137* | 1SM7 | 7065 | 2G9L |
| 10196 | 2EM5 | 11237 | 2YSM | 15323 | 2JR8 | 16214 | 2KGO | 17110 | 2L21 | 18002 | 2LKO | 18818 | 2M0R | 19710 | 2MJ8 | 25817 | 2N7Q | 5174 | 1IRZ | 6138 | 1T0G | 7070 | 2GO9 |
| 10197 | 2EM6 | 11238 | 2YRH | 15324 | 2JRA | 16215 | 2LY5 | 17111 | 2L22 | 18003 | 2LKP | 18822 | 2M0S | 19712 | 2MJ9 | 25850 | 2N8I | 5177 | 1TVJ | 6139 | 1SS3 | 7074 | 2GMG |
| 10198 | 2EM8 | 11240 | 2COO | 15325 | 2JRB | 16219 | 2KGT | 17124 | 2L25 | 18004 | 4A24 | 18830 | 2M0W | 19714 | 2MN6 | 25857 | 2N8O | 5178 | 1K85 | 6150 | 2POA | 7075 | 2GPF |
| 10199 | 2EMA | 11244 | 2EQR | 15327 | 2HGK | 16231 | 2KHA | 17127 | 2L29 | 18006 | 4A4F | 18832 | 2M0Y | 19716 | 2MJC | 25858 | 2N8P | 5179 | 1K1Z | 6151 | 1SMZ | 7078* | 2AIH |
| 10200 | 2EMB | 11245 | 1UH6 | 15332 | 2JRH | 16233 | 2LBO | 17132 | 2L2D | 18009 | 2LKQ | 18840 | 2M13 | 19717 | 2MJD | 25864 | 2N8S | 5181 | 1K45 | 6159 | 2B1O | 7079 | 2GQB |
| 10201 | 2EMC | 11246 | 1WLN | 15334 | 2JRL | 16236 | 2KHC | 17138 | 2L2L | 18011 | 2LVV | 18841 | 3ZBE | 19729 | 2MJK | 25865 | 2N8T | 5184 | 1LGL | 6161 | 1UST | 7085 | 2GRG |
| 10202 | 2EMH | 11247 | 2CSW | 15338 | 2JRP | 16238 | 2KHD | 17143 | 2L2N | 18012 | 2LKT | 18843 | 2M74 | 19731 | 2MJL | 25868 | 2N8W | 5185 | 1KAT | 6165 | 1SSL | 7087 | 2H0P |
| 10204 | 2EMJ | 11248 | 2YUB | 15339 | 2JRM | 16247 | 2KHK | 17145 | 2L7M | 18014 | 2LKW | 18850 | 2M19 | 19732 | 2MJM | 25907 | 2N9I | 5187 | 1V4R | 6167 | 1SNL | 7092 | 2GL1 |
| 10205 | 2EMK | 11250 | 2RRE | 15341 | 2JRR | 16248 | 2KFU | 17149 | 2L2P | 18016 | 2LKY | 18853 | 2MHK | 19735 | 2MJN | 25922 | 2N9Z | 5189 | 1NSH | 6172 | 1SB6 | 7093 | 2GTV |
| 10206 | 2EOH | 11251 | 2RRF | 15343 | 2JRS | 16249 | 2KHM | 17150 | 2L2Q | 18017 | 2LKZ | 18856 | 2M1C | 19737 | 2MJU | 25933 | 2NAA | 5192 | 1K8V | 6173 | 2LP6 | 7095 | 2GOW |
| 10207 | 2EOW | 11253 | 1V5O | 15347 | 2K8S | 16250 | 2KHN | 17152 | 2L2R | 18019 | 2LL1 | 18865 | 2M1J | 19749 | 2MK2 | 25934 | 2NAB | 5194 | 1K5W | 6175 | 1SZL | 7097 | 2GLO |
| 10208 | 2EOX | 11254 | 1V6E | 15348 | 2JRZ | 16252 | 2L7Z | 17160 | 2LHS | 18022 | 2LL2 | 18869 | 2M1L | 19750 | 2MK3 | 25953 | 2NAX | 5199 | 1KG1 | 6176 | 1T0Y | 7099 | 2GJI |
| 10209 | 2EOZ | 11255 | 1V95 | 15349 | 2JS0 | 16253 | 2KHR | 17161 | 2L9B | 18043 | 2LLD | 18870* | 2M1M | 19751 | 2MK4 | 25995 | 2NBV | 5203 | 1KBE | 6177 | 1SJR | 7102 | 2GJF |
| 10210 | 2EP0 | 11256 | 1WGG | 15350 | 2JS1 | 16254 | 2KHT | 17175 | 2L3A | 18044 | 2LLE | 18877 | 2M29 | 19752 | 2MK5 | 26517 | 5AGQ | 5210 | 1K7B | 6185 | 1RZS | 7106 | 2JM5 |
| 10212 | 2CUD | 11257 | 1WGH | 15351 | 2JS2 | 16256 | 2KHX | 17176 | 2L3B | 18047 | 2LLG | 18880 | 2M1U | 19753 | 2N03 | 26550 | 2N1F | 5213 | 1K0V | 6187 | 1SF0 | 7108 | 2GZY |
| 10213 | 2E29 | 11259 | 1WGL | 15352 | 2JS3 | 16258 | 2KHZ | 17194 | 2L3I | 18051 | 2LLK | 18882 | 2M1W | 19755 | 2MK6 | 26553 | 5AJ1 | 5217 | 1KFT | 6188 | 1ST7 | 7112 | 2HWT |
| 10214 | 2YSE | 11260 | 1WGN | 15353 | 2JS4 | 16260 | 2KI3 | 17196 | 2L3M | 18053 | 2LLL | 18883 | 2M1X | 19764 | 2MK9 | 26580 | 5A3G | 5220 | 1K0X | 6190 | 1SP0 | 7114 | 2H25 |
| 10217 | 2EOG | 11261 | 1WX7 | 15354 | 2JS5 | 16263 | 2KM6 | 17199 | 2L3N | 18083 | 2LLP | 18887 | 2M1Z | 19765 | 2MKB | 26674* | 2N8H | 5225 | 1JNS | 6197 | 2BTT | 7116* | 2GLW |
| 10218 | 2EOJ | 11262 | 1WX8 | 15361 | 2JSD | 16268 | 2KI9 | 17200 | 2L3R | 18085* | 2LLR | 18896 | 2N17 | 19766 | 2MKC | 34014 | 5LCS | 5232 | 1KXL | 6198 | 1T3V | 7117 | 2H1Z |
| 10220 | 2EON | 11263 | 1WX9 | 15370 | 2JSN | 16271 | 2KIE | 17202 | 2L3T | 18086 | 2LLZ | 18900 | 3ZG4 | 19783 | 2MKL | 4020 | 1BRV | 5238 | 1VGH | 6200 | 1SP7 | 7120 | 2GYT |
| 10221 | 2EOR | 11264 | 1WXA | 15371 | 2JSO | 16272 | 2KIF | 17203 | 2N57 | 18090 | 2LLV | 18901 | 2M26 | 19797 | 2MKV | 4090 | 2EZH | 5241 | 1RQM | 6208 | 1TUZ | 7121 | 2DO8 |
| 10223 | 2YTR | 11265 | 1WXM | 15373 | 2JSP | 16273 | 2KIG | 17232 | 2L47 | 18091 | 2LLW | 18904 | 2M2A | 19799 | 2MKX | 4114 | 1A5J | 5242 | 1KN6 | 6209 | 1TIZ | 7122 | 2G7H |
| 10224 | 2EMV | 11266 | 1WY8 | 15374 | 2QL0 | 16276 | 2KII | 17233 | 2L48 | 18093 | 2LLY | 18906 | 2N5N | 19800 | 2MKY | 4141 | 1NK2 | 5255 | 1WKT | 6211 | 1TDP | 7124 | 2DQ5 |
| 10225 | 2EMW | 11268 | 1X1M | 15377 | 2JR7 | 16277 | 2KIJ | 17234 | 2L4A | 18094 | 2LM0 | 18908 | 2M2D | 19806 | 2ML5 | 4147 | 1H95 | 5257 | 1HJ0 | 6216 | 1SRK | 7125 | 2M6Z |
| 10226 | 2EMX | 11269 | 2DAE | 15381 | 2JSX | 16293 | 2KIS | 17235 | 2L4B | 18095 | 2LM1 | 18909 | 2M2E | 19808* | 2ML7 | 4148 | 4BMF | 5261 | 1KTU | 6221 | 1XSF | 7127 | 2H45 |
| 10227 | 2EMY | 11270 | 2DAF | 15382 | 2JOT | 16297 | 2KIV | 17241 | 2L4J | 18097 | 2LM3 | 18910 | 3ZGK | 19809 | 2ML9 | 4157 | 1LFC | 5264 | 1KJ6 | 6222 | 1T1T | 7129 | 2GGR |
| 10228 | 2EMZ | 11271 | 2DAH | 15386 | 2JT1 | 16298 | 2KIW | 17242 | 2L4Q | 18098 | 2LM4 | 18912 | 2M2F | 19810 | 2MLA | 4177 | 1BO0 | 5265 | 1KV4 | 6226 | 2RST | 7137 | 2H7T |
| 10229 | 2EN0 | 11272 | 2DAJ | 15390 | 2JRD | 16311 | 2KJ4 | 17244 | 2L43 | 18107 | 2LM9 | 18916 | 2M2I | 19811 | 2MLB | 4181 | 1PFD | 5268* | 1CIX | 6231 | 2GO0 | 7151 | 2H80 |
| 10231 | 2EN2 | 11273 | 2DAL | 15393 | 2JSS | 16312 | 2KJ5 | 17245 | 2L4N | 18108 | 2LME | 18917 | 2M2J | 19821 | 2MLG | 4186 | 1BLR | 5272* | 1KGM | 6233 | 1YX6 | 7158 | 2GQE |
| 10232 | 2EN3 | 11274 | 2DAM | 15405 | 2JOO | 16315 | 2KJ8 | 17246 | 2L4O | 18111 | 2LMC | 18920 | 2M2L | 19823 | 2MLJ | 4188 | 1BCI | 5275 | 1KRI | 6236 | 1TKN | 7166 | 2H9X |
| 10233 | 2EN4 | 11275 | 2DHX | 15407* | 2JTE | 16316 | 2KJ9 | 17247 | 2KN0 | 18112 | 2LMD | 18926 | 2M2Q | 19824 | 4CRP | 4190* | 1BIG | 5294 | 1K81 | 6238 | 1TOT | 7167 | 2I3E |
| 10241 | 1X6B | 11276 | 2DHY | 15411 | 2JSW | 16317 | 2KLL | 17249 | 2ML2 | 18113 | 2MBX | 18939 | 2MJG | 19828 | 2MLO | 4191* | 2BMT | 5297 | 1L2M | 6241 | 1YZB | 7178 | 2GZP |
| 10242 | 1X6D | 11277 | 2DHZ | 15415 | 2JTM | 16319 | 2KJF | 17252 | 2KN1 | 18115 | 2LMG | 18942 | 2M32 | 19834 | 2MLW | 4195 | 2BTX | 5304 | 1KX7 | 6244 | 1TM9 | 7180 | 2GZO |
| 10243 | 1X6E | 11278 | 2DI0 | 15419 | 2JTV | 16320 | 2KJG | 17256 | 2L4U | 18125 | 2MF6 | 18943 | 3ZFJ | 19840 | 2MM0 | 4197 | 2CPS | 5308 | 1M7T | 6247 | 1TH5 | 7181 | 2H3J |
| 10244 | 1X6F | 11279 | 2DZI | 15423 | 2JTY | 16325 | 2KJK | 17258 | 2L4V | 18126 | 2LR2 | 18945 | 2M35 | 19841 | 2MM2 | 4198 | 1BR0 | 5309 | 1M3V | 6248 | 1TTV | 7182 | 2H7A |
| 10245 | 1X6G | 11280 | 2DZJ | 15430 | 2JU4 | 16335 | 2KJP | 17260 | 2LEN | 18130 | 2LRC | 18946 | 2M36 | 19845 | 2MM4 | 4200 | 1A93 | 5310 | 2PLD | 6254 | 1TXE | 7185 | 2GUT |
| 10246 | 1X6H | 11281 | 2DZK | 15431 | 2JU5 | 16338 | 2KJR | 17263 | 2L52 | 18134 | 2LMR | 18951 | 2M3A | 19846* | 2MM5 | 4202 | 1BA9 | 5312 | 1NEE | 6259 | 1TVM | 7186 | 2GX1 |
| 10247 | 2CO8 | 11282 | 2DZL | 15437 | 2JUA | 16339 | 2KJL | 17265 | 2L4Z | 18135 | 2LMS | 18953 | 2M3C | 19847* | 2MM6 | 4206 | 1MFN | 5315 | 1L4S | 6261 | 1W1F | 7189 | 2GI4 |
| 10248 | 1WZ6 | 11283 | 2DZM | 15438 | 2JUB | 16344 | 2KJV | 17266 | 2L55 | 18141 | 2LMZ | 18955 | 3ZJ1 | 19848 | 4CSQ | 4208 | 2DVH | 5316 | 1L6N | 6262 | 1TK7 | 7201 | 2HJQ |
| 10249 | 1X05 | 11284 | 1WXT | 15439 | 2JUC | 16345 | 2KJW | 17267 | 2L57 | 18142 | 2LN0 | 18956 | 3ZJ2 | 19849 | 2MM9 | 4211 | 1BBG | 5323 | 1L1I | 6263 | 1SPW | 7205 | 2LCX |
| 10250 | 1X1F | 11285 | 1WXU | 15440 | 2M2K | 16349 | 2KK1 | 17270 | 2L5E | 18145 | 2LN3 | 18964 | 2M3I | 19850 | 2MMA | 4212 | 1BCV | 5326 | 1L6T | 6264 | 1TV0 | 7209 | 2IVW |
| 10251 | 1X1G | 11286 | 1X3A | 15442 | 2JUG | 16350 | 2KK2 | 17271 | 2L5G | 18146 | 2LN4 | 18966 | 2M3K | 19856 | 2MMJ | 4214 | 2TMP | 5328 | 1L7B | 6265 | 1T1H | 7220 | 2I85 |
| 10252 | 2CO9 | 11287 | 1X3C | 15444 | 2JUH | 16352 | 2KK4 | 17275 | 2L5L | 18153 | 2LN8 | 18967 | 2M3L | 19859 | 2MMM | 4217 | 1BHU | 5329 | 1L7Y | 6269 | 1U3M | 7224 | 2HG7 |
| 10253 | 2COA | 11288 | 1X3D | 15449 | 2PNG | 16353 | 2KK6 | 17277 | 2L5O | 18156 | 2LNA | 18970 | 2M3N | 19860 | 2MMP | 4218 | 1CN2 | 5333 | 1IT1 | 6272 | 1Z9V | 7225 | 2HEP |
| 10254 | 2COC | 11289 | 1X3H | 15451 | 2JUO | 16354 | 2KK7 | 17278 | 2MOT | 18158 | 2LNB | 18972* | 3ZKT | 19866 | 2ND4 | 4221* | 1BM4 | 5335 | 1LV3 | 6282 | 2MMN | 7226 | 2HGA |
| 10255 | 2COD | 11290 | 1X5X | 15456 | 2JUW | 16355 | 2KK8 | 17279 | 2L5P | 18159 | 2YOM | 18986 | 2M45 | 19867 | 2MMU | 4224 | 1MYO | 5338 | 1L3Y | 6284 | 2HAJ | 7228 | 2HI6 |
| 10256 | 2COF | 11291 | 1X5Y | 15457 | 2KBB | 16357 | 2KKE | 17281 | 2L5R | 18160 | 2YON | 18987 | 2M46 | 19869 | 2MMV | 4225 | 3GRX | 5345 | 1L1M | 6285 | 1U89 | 7229 | 2HDL |
| 10257 | 2DJR | 11292 | 1X5Z | 15462 | 2JUZ | 16361 | 2KKC | 17283 | 2LJ6 | 18161 | 2LND | 18989 | 2M47 | 19872 | 2MMZ | 4230 | 1BUQ | 5346 | 1L3X | 6286 | 1XC5 | 7256 | 2HFD |
| 10258 | 2DJS | 11293 | 2CU8 | 15466 | 2JV4 | 16365 | 2KKM | 17284 | 2L5T | 18164 | 2LNG | 19000 | 2M4E | 19875 | 2MPC | 4241 | 2XBD | 5348 | 1IW4 | 6295 | 1TVC | 7257 | 2HVZ |
| 10259 | 2DJT | 11294 | 2DAW | 15471 | 2JVA | 16366 | 2KKN | 17287 | 2LLA | 18166 | 2LNI | 19001 | 2M4F | 19876 | 2MN2 | 4246 | 2V1V | 5349 | 1LFU | 6298 | 1TEY | 7258* | 2HN8 |
| 10261 | 2DJV | 11295 | 2DAX | 15476 | 2JVD | 16368 | 2KKO | 17288 | 2MF2 | 18167 | 2LNJ | 19002 | 2M4G | 19878 | 2MN3 | 4248 | 2LEF | 5354 | 1LL8 | 6299 | 1U5M | 7259 | 2HTF |
| 10262 | 2DKM | 11297 | 2DIG | 15477 | 2JVE | 16370 | 2KKQ | 17289 | 2L5Y | 18170 | 2LNL | 19003 | 2M4H | 19879 | 2MN4 | 4249 | 1XPA | 5356 | 1LQ7 | 6300 | 1U3O | 7260 | 2HGC |
| 10263 | 2DN7 | 11300 | 2DIM | 15478 | 2JVF | 16373 | 2KKV | 17291 | 2L60 | 18171 | 2LNM | 19007 | 2M4I | 19880 | 2MN5 | 4254 | 1L3G | 5363 | 1LO1 | 6302 | 1U7M | 7261 | 2HJJ |
| 10264 | 1X5B | 11301 | 2DIN | 15491 | 2JVW | 16377 | 2LEK | 17298 | 2MDT | 18180 | 2LNU | 19013 | 2M4M | 19893 | 2MNI | 4255 | 2IF1 | 5364 | 1GO5 | 6303 | 1U7J | 7262 | 2JM2 |
| 10265 | 2EDX | 11302 | 2DMD | 15504 | 2JW1 | 16378 | 2KL1 | 17304 | 2L69 | 18181 | 2LNV | 19014 | 2M4N | 19906 | 2MNU | 4257* | 1VPC | 5366 | 1KMD | 6311 | 2AFJ | 7263 | 2I1P |
| 10266 | 2EDY | 11304 | 2DOC | 15507 | 2VCD | 16380 | 2KL2 | 17305 | 2L6A | 18182 | 2LNW | 19023 | 2M4V | 19910 | 2MNW | 4264 | 1VRC | 5375* | 1LD6 | 6312 | 1W7D | 7266 | 2HFQ |
| 10267 | 2EDZ | 11305 | 1V6G | 15509 | 2JUF | 16381 | 2K4T | 17306 | 2L6B | 18184 | 2LNY | 19027 | 2M4Y | 19939 | 2MOE | 4265 | 1DS9 | 5383 | 1LV9 | 6315 | 1U6F | 7268 | 2V37 |
| 10268 | 2EE0 | 11307 | 1WIM | 15511 | 2JW8 | 16386 | 2KL7 | 17312 | 2L6J | 18186 | 2LNZ | 19042 | 2MPE | 19941 | 2MOF | 4269 | 1GHT | 5390 | 1LR1 | 6317 | 2GZU | 7269 | 2JNK |
| 10269 | 2EE1 | 11308 | 1WIR | 15512 | 2LQR | 16388 | 2KLB | 17315 | 2L6M | 18187 | 2LO0 | 19045 | 2M5B | 19942 | 2MOG | 4292 | 1B1V | 5392 | 1L8Y | 6318 | 1XFL | 7270 | 2O4E |
| 10270 | 2EE2 | 11309 | 1WJV | 15528 | 2JWN | 16389 | 2KLA | 17318 | 2L6N | 18191 | 2LO2 | 19056 | 2MOQ | 19945 | 2MOK | 4334 | 1KQQ | 5399 | 1SXE | 6325 | 1WO7 | 7271 | 2NNZ |
| 10271 | 2EE3 | 11310 | 1WJW | 15536 | 2JWT | 16390 | 2KLC | 17319 | 2L6P | 18193 | 2LQK | 19059 | 2M5L | 19947 | 2MOP | 4347 | 1QP6 | 5403 | 1C9S | 6333 | 2XV9 | 7272 | 2I59 |
| 10272 | 2EKH | 11311 | 1X3B | 15542 | 2JWY | 16396 | 2KLQ | 17320 | 2L6O | 18194 | 2LT1 | 19066 | 3ZPD | 19952 | 2MOU | 4351 | 1QGM | 5410* | 1MPZ | 6335 | 1XJH | 7273 | 2I1T |
| 10274 | 2EKJ | 11312 | 1X5W | 15543 | 2JX2 | 16397 | 2KKR | 17324 | 2MC3 | 18195 | 2LO4 | 19067 | 3ZPM | 19955 | 2MOX | 4373 | 1FHO | 5459 | 1IYM | 6338 | 2EVN | 7274 | 2HH8 |
| 10275 | 2YU0 | 11313 | 2CSY | 15547 | 2JX5 | 16400 | 2KLW | 17327 | 2L6X | 18200 | 2MLK | 19070 | 2M5P | 19959 | 4CYK | 4378 | 2LCP | 5461 | 1LUI | 6339 | 1XO8 | 7276 | 2MEY |
| 10276 | 2D9T | 11314 | 2CSZ | 15550 | 2JX8 | 16403 | 2KLX | 17329 | 2L4H | 18201 | 2M5J | 19077 | 2MUU | 19960 | 2MP1 | 4387* | 8TFV | 5464 | 1M36 | 6341 | 1XOY | 7287 | 2I7K |
| 10277 | 2D9U | 11315 | 2CT0 | 15553 | 2JX9 | 16404 | 2KLY | 17343 | 2L73 | 18203 | 2LO9 | 19079 | 2M5T | 19966 | 2N34 | 4391* | 1JGK | 5465 | 1M12 | 6342 | 1XOX | 7291 | 2NPL |
| 10278 | 2D9V | 11316 | 2CT1 | 15555 | 2JXD | 16405 | 2KLZ | 17352 | 2L7E | 18210 | 2LOE | 19087 | 2M5Y | 19970 | 2MP8 | 4395 | 1B75 | 5467 | 1RI9 | 6343 | 1XPN | 7297 | 2IDA |
| 10279 | 2D9W | 11317 | 2CT2 | 15559 | 2JXF | 16411 | 2KM4 | 17353 | 2LMT | 18211 | 2LY2 | 19094 | 2M5Z | 19971 | 2MXA | 4396 | 1COU | 5475 | 1M9W | 6344 | 1XPW | 7298 | 2I50 |
| 10280 | 2D9X | 11318 | 2CT4 | 15562 | 2JXN | 16413 | 2LJK | 17355 | 2L9F | 18214 | 2LOJ | 19095 | 2MNA | 19977 | 2MPF | 4397 | 1B2T | 5481 | 1M94 | 6347* | 1WPD | 7299 | 2J5H |
| 10281 | 2CUE | 11319 | 2CT5 | 15574 | 2JXU | 16425 | 2KM8 | 17358 | 2L7J | 18215 | 2LOK | 19098 | 2MZJ | 19986 | 2MPJ | 4405 | 1KHM | 5482 | 1MJD | 6354 | 1TQZ | 7301 | 2O3D |
| 10282 | 2CUF | 11320 | 2CT6 | 15575 | 2JXW | 16426 | 2KMD | 17359 | 2L7K | 18216 | 2LOL | 19101 | 2M60 | 19988 | 2MPL | 4407 | 1CMZ | 5489* | 1MA6 | 6355 | 1XHJ | 7302 | 2I4K |
| 10283 | 2DA1 | 11321 | 2CT7 | 15576 | 2JXX | 16428 | 2KMG | 17363 | 2L7N | 18221 | 2LOQ | 19106 | 2M63 | 19994 | 2MPN | 4413 | 1COK | 5491* | 1IYC | 6356 | 2GA5 | 7313 | 2IUE |
| 10284 | 2DA2 | 11322 | 2CU7 | 15579 | 2JY0 | 16442 | 2KMW | 17365 | 2L7P | 18222 | 2LOR | 19107 | 2M64 | 19998 | 2MPQ | 4414 | 1B3I | 5498 | 1K4U | 6358 | 1XS3 | 7320 | 2NRG |
| 10285 | 2DA3 | 11323 | 2D8Q | 15584 | 2JY9 | 16452* | 2KN8 | 17370 | 2L7Q | 18223 | 2LOS | 19108 | 2M65 | 20048* | 2LXG | 4417 | 1B6F | 5499 | 1M5Z | 6362 | 1XJS | 7322 | 2NLN |
| 10286 | 2DA4 | 11324 | 2D8R | 15585 | 2JYA | 16472 | 2KNO | 17371 | 2L7R | 18228 | 2M3V | 19111 | 2MUX | 21019 | 2RSW | 4422* | 1CCV | 5501 | 1M4F | 6365 | 1XN6 | 7323 | 2NO8 |
| 10287 | 2DA5 | 11325 | 2D8S | 15596 | 2JYO | 16477 | 2KNU | 17374 | 2LJ8 | 18242 | 2M5V | 19124 | 2M6A | 25002 | 2MPW | 4425 | 3BDO | 5506 | 1NWV | 6366 | 1ZTS | 7324 | 2NPB |
| 10288 | 2DA6 | 11326 | 2D8T | 15601 | 2KPH | 16478 | 2KNA | 17381 | 2L7Y | 18244 | 2LPB | 19126 | 2M6B | 25007 | 2MQ0 | 4427 | 1QKY | 5516* | 1RSF | 6367 | 1XN7 | 7327 | 2NSW |
| 10289 | 2DA7 | 11327 | 2D8U | 15604 | 2JZ2 | 16480 | 2LGP | 17382 | 2L7W | 18255 | 2LPD | 19142 | 2M6J | 25008 | 2MQ1 | 4429 | 1RQU | 5525 | 1M9O | 6369 | 1XN5 | 7330 | 2E0H |
| 10290 | 2DJN | 11328 | 2D8V | 15607 | 2JZ4 | 16484 | 2KNZ | 17383 | 2L7X | 18277 | 2LPU | 19143 | 2M6K | 25024 | 2MQA | 4430 | 2CNP | 5527 | 1MB6 | 6376 | 2BVB | 7341 | 2J15 |
| 10291 | 2DMN | 11329 | 2DAT | 15608 | 2JZ5 | 16485 | 2KO0 | 17388 | 2L80 | 18278 | 2LPV | 19146 | 2M6M | 25028 | 2MQD | 4437 | 1CEJ | 5532 | 1M4P | 6382 | 1XU0 | 7352 | 2BL6 |
| 10292 | 2DMP | 11331 | 2DJA | 15609 | 2JZ6 | 16486 | 2KO1 | 17389 | 2L81 | 18281 | 2LPX | 19147 | 2M6N | 25030 | 2MQE | 4447 | 1H7Y | 5535 | 1MSZ | 6384 | 1XUT | 7362 | 2NWT |
| 10293 | 2DMQ | 11332 | 2DJB | 15610 | 2JZ8 | 16490 | 2KO6 | 17390 | 2L82 | 18283 | 2LRV | 19148 | 2M6O | 25033 | 2MQH | 4452 | 2AFP | 5538 | 1H3Z | 6386* | 1Y1B | 7365 | 2E45 |
| 10294 | 2DMS | 11333 | 2DKT | 15611 | 2JZA | 16492 | 2KO8 | 17391 | 2L83 | 18284 | 2LQ0 | 19155 | 2M6U | 25036 | 2MQJ | 4455 | 1CI5 | 5545 | 1RMJ | 6388 | 1XV3 | 7371 | 2OA4 |
| 10295 | 2DMT | 11334 | 2DME | 15614 | 2JZB | 16496 | 2KNC | 17395 | 2L87 | 18286 | 2LQ1 | 19162 | 2M6X | 25037 | 2MQK | 4461 | 1DF6 | 5551 | 1H0Z | 6389* | 1WT7 | 7382 | 2OSR |
| 10296 | 2DMU | 11335 | 2EA5 | 15617 | 2JZC | 16498 | 2KOB | 17396 | 2L8E | 18289 | 2LQ2 | 19167 | 2M70 | 25048 | 2MQS | 4486 | 2MOB | 5552 | 1M3G | 6390 | 2AGM | 7395 | 2YZ0 |
| 10297 | 2E19 | 11336 | 2EA6 | 15622 | 2JOB | 16504 | 2KOE | 17398 | 2L89 | 18290 | 2LQ3 | 19168 | 2M71 | 25050 | 2MQU | 4487 | 1CE3 | 5557 | 1MM4 | 6395 | 1YR1 | 7401 | 2P6J |
| 10298 | 2YS9 | 11337 | 2EBK | 15632 | 2JZY | 16507 | 2KOH | 17399 | 2L8A | 18297 | 2LQ7 | 19169 | 2M72 | 25052 | 2MQV | 4491 | 1QJT | 5560 | 1RDU | 6398 | 1Y9O | 7402 | 2PCO |
| 10299 | 2DB8 | 11338 | 2EBM | 15634 | 2K02 | 16515 | 2KOJ | 17402 | 2L8D | 18298 | 2LQ8 | 19170 | 2M73 | 25061 | 2MR5 | 4492 | 1JBA | 5561 | 1O6X | 6399 | 1HH8 | 7419 | 2V6Z |
| 10300 | 2DBJ | 11339 | 2ECG | 15638 | 2N48 | 16517 | 2KOK | 17403 | 3ZUA | 18300 | 2LQA | 19174 | 2M76 | 25062 | 2MR6 | 4496 | 1OM2 | 5564 | 1MZK | 6407 | 2FK4 |  |  |
| 10302 | 2DBM | 11340 | 2ECI | 15644 | 2K0A | 16521 | 2KON | 17405 | 4AKA | 18301 | 2LQB | 19178 | 2M7A | 25066 | 2MR9 | 4497 | 7HSC | 5565 | 1XHH | 6410 | 1Z7R |  |  |
| 10303 | 2EML | 11341 | 2ECJ | 15645 | 2K0D | 16534 | 2KOU | 17407 | 2LP0 | 18313 | 2LQG | 19179 | 2M7B | 25067 | 2MRA | 4500 | 1CNN | 5570 | 1Q27 | 6416 | 1UIV |  |  |
| 10304 | 2EMM | 11342 | 2ECL | 15651 | 2K0L | 16536 | 2KOY | 17412 | 2L8J | 18316 | 2LQJ | 19185 | 2M7G | 25068 | 2MRC | 4503 | 1QMW | 5576 | 1MO8 | 6419 | 1XU6 |  |  |
| 10305 | 2EMP | 11343 | 2ECM | 15652 | 2K0M | 16545 | 2KP6 | 17418 | 2LCJ | 18318 | 2LQL | 19187 | 2MA3 | 25071 | 2MRF | 4510 | 1CXW | 5582 | 1MQY | 6434 | 1Z1Z |  |  |
| 10306 | 2YRM | 11344 | 2ECN | 15655 | 2K0Q | 16556 | 2KPI | 17420 | 2L8O | 18320 | 2LQM | 19195 | 2M7L | 25079 | 2MV6 | 4514 | 1D2L | 5583 | 1L3H | 6438 | 2J48 |  |  |
| 10307 | 2YTD | 11345 | 2ELJ | 15660 | 2M1S | 16557 | 2KPJ | 17424 | 2L8S | 18321 | 2LQN | 19200 | 2M7P | 25083 | 2MRL | 4519 | 1D7Q | 5585* | 1MTQ | 6443 | 1YDU |  |  |
| 10308 | 2YTE | 11346 | 2RPJ | 15663 | 2LC5 | 16558 | 2KPK | 17429 | 2L8V | 18322 | 2LQO | 19201 | 2M7Q | 25086 | 2MRN | 4540 | 1NLA | 5589 | 1NXI | 6447 | 1Y7X |  |  |
| 10309 | 2YTJ | 11347 | 2YQL | 15665* | 2K10 | 16560 | 2KPM | 17431 | 2L8Y | 18328 | 2LQT | 19205 | 2M7T | 25096 | 2MRW | 4541 | 1QUZ | 5596 | 1N91 | 6448 | 1XHS |  |  |

*Entries used in the calibration at acidic pH

| **Table S3. BMRB entries used in the parameterization of PROSECCO*_FOLDED_***. **of proteins not having a deposited structure in the PDB.** | | | | | | | | | | | | | | | | | | | | | | | | |
| --- | --- | --- | --- | --- | --- | --- | --- | --- | --- | --- | --- | --- | --- | --- | --- | --- | --- | --- | --- | --- | --- | --- | --- | --- |
| 10004 | 11484 | 1518 | 15557 | 15877 | 16305 | 16718 | 17195 | 17608 | 18165 | 18682 | 19172 | 19736 | 25059 | 25600 | 300 | 4161 | 4438 | 4848 | 5227 | 5680 | 6069* | 6428 | 6777 | 7123 |
| 10005 | 11487 | 15180 | 15560 | 15878 | 16307 | 16729 | 17201 | 17616 | 18177 | 18689 | 1918 | 19740 | 25063 | 25619 | 306 | 4162 | 4444 | 4849 | 5228 | 5682 | 6074 | 6429 | 6782 | 7133 |
| 10010 | 11488 | 15193 | 15561 | 1588* | 16313 | 1673 | 17204 | 17620 | 18179 | 18691 | 19182 | 19757 | 25065 | 25624 | 321 | 4163 | 4449 | 4851 | 5233 | 5685 | 6078 | 6432 | 6783 | 7149* |
| 10012 | 11494 | 15194 | 15566 | 15881 | 16321 | 16733 | 17205 | 17630 | 18188 | 18693 | 19191 | 19758 | 25069 | 25626 | 337 | 4168 | 4451 | 4852* | 5234 | 5686 | 6086 | 6433 | 6784 | 7150 |
| 10023 | 11516 | 15204 | 15570 | 15883* | 16322 | 16734 | 17206 | 17634 | 18189 | 18696 | 19194 | 19768 | 25077 | 25640 | 3394 | 4171 | 4453 | 4853 | 5237 | 5687 | 6093 | 6436 | 6785 | 7161 |
| 10052* | 11522 | 15207 | 15577 | 15885 | 16323 | 16735 | 17210 | 17639 | 18196 | 18701 | 19204 | 19774 | 25081 | 25665 | 3427 | 4185* | 4457 | 4856 | 5244 | 5692 | 6096* | 6439 | 6787 | 7162 |
| 10053 | 11526 | 15213 | 15581 | 15892 | 16329 | 16738 | 17211 | 17640 | 18197 | 18704 | 19206 | 19787 | 25087 | 25666 | 3433 | 4193 | 4459 | 4857 | 5263 | 5699 | 6097 | 6441 | 6793 | 7165 |
| 10061 | 11546 | 15214 | 15587 | 15897 | 16337 | 16739 | 17212 | 17643 | 18198 | 18718 | 19208 | 19788 | 25108 | 25674 | 3437 | 420 | 4460 | 4859 | 5267 | 5704 | 6098 | 6451 | 6796 | 7172 |
| 10062 | 1155 | 15217 | 15590 | 15903 | 16340 | 16741 | 17226 | 17650 | 18202 | 18719 | 19209 | 19796 | 25109* | 25685 | 3456 | 421* | 4462 | 4871 | 5269 | 5707 | 6099 | 6452 | 6799 | 7173 |
| 10078 | 11574 | 15222 | 15593 | 15911 | 16341 | 16744 | 17231 | 17656 | 18220 | 18731 | 19212 | 19801 | 25119 | 25697 | 3466 | 4228 | 4463 | 4876 | 5274* | 5709 | 6112 | 6453 | 6806 | 7175 |
| 10096 | 11575 | 15225 | 15595 | 15914 | 1635 | 16748 | 17239 | 17663 | 18225 | 18738 | 19215 | 19818 | 25124 | 257 | 3485* | 4231 | 4465 | 4881 | 5280 | 5736 | 6117 | 6460 | 6820 | 7176 |
| 10145 | 11584 | 15241 | 15597 | 15924 | 16359 | 1675 | 17250 | 17664 | 18229 | 18741 | 19236 | 19826 | 25131 | 25715 | 349 | 4236 | 4466 | 4885 | 5288 | 5741 | 6118 | 6469 | 6823 | 7179 |
| 10190 | 11588 | 15243 | 15606 | 15933 | 16375 | 16766 | 17251 | 17672 | 18235 | 18748 | 19239 | 19831 | 25138 | 25773 | 3548 | 4237 | 447 | 4896 | 5299 | 5749* | 6125 | 6473* | 6824 | 7187 |
| 10203 | 1163 | 15248 | 15612 | 15937* | 16379 | 16769 | 17254 | 17681 | 18236 | 18758 | 19243 | 19833 | 25146 | 2580 | 356 | 4251 | 4470 | 4897 | 5300 | 5751 | 6132 | 6476 | 6825 | 7188 |
| 10216 | 1164 | 15249 | 15615 | 15940 | 16395 | 16770 | 1727 | 17695 | 18246 | 18760 | 19267 | 19844 | 25154 | 25802 | 358* | 4259 | 4471 | 4898 | 5311 | 5755 | 6133 | 6494 | 6828 | 7191 |
| 10222 | 1165 | 15256 | 15623 | 15942 | 16398 | 16773 | 17273 | 17711 | 18248 | 18761 | 19271 | 19854 | 25158 | 25804 | 367 | 4267 | 4477* | 4901 | 5322 | 5756 | 6136 | 65* | 6832 | 7192 |
| 10230 | 1167 | 15263 | 15625 | 15954 | 16402 | 16780 | 17276 | 17742 | 18249 | 18775 | 19272 | 19885 | 25161 | 25827 | 372* | 4273 | 448 | 4913 | 5325 | 5760 | 6148 | 6501 | 6834 | 7193 |
| 10239 | 1168 | 15264 | 15627 | 15957* | 16424 | 16781 | 1728* | 17743 | 1825 | 18779 | 19296 | 19901 | 25162 | 25838 | 374 | 4279 | 4559 | 4918 | 5343 | 5761 | 6149 | 6502 | 6836 | 7202 |
| 1037 | 1198* | 15266 | 15628 | 15959 | 16430 | 16784* | 17285 | 17748 | 18250 | 18791 | 19297 | 19904 | 25166 | 25849 | 376 | 428 | 4560 | 4926 | 5350 | 5763 | 6156 | 6504 | 6837 | 7203 |
| 1061 | 1203 | 15271 | 15637 | 15963 | 16435* | 16788 | 1729* | 17755 | 18257 | 18803 | 19301 | 19914 | 25173 | 25873 | 387 | 4280 | 4561* | 4928 | 5352 | 5772 | 6157 | 6508 | 6838 | 7204 |
| 1065 | 132 | 15276 | 15639 | 15964 | 16439 | 16798 | 17290 | 17760 | 18260 | 18815 | 19306 | 19916 | 25179 | 25893 | 390 | 4282 | 4562 | 4932 | 5355 | 5774 | 6171 | 6512 | 6840 | 7206 |
| 1071 | 133 | 15278 | 15640 | 15968 | 16441 | 16803 | 17293 | 17761 | 18267 | 18820 | 19318 | 19921 | 25182 | 25895 | 397* | 4286 | 4565 | 4933 | 5360 | 5778 | 6180 | 6515 | 6847 | 7207 |
| 11008 | 1336 | 15280 | 15642 | 15969 | 16446 | 16804 | 17295 | 17766 | 18280 | 18823 | 19322 | 19928 | 25183 | 25897 | 398 | 4289 | 4571 | 4937 | 5362 | 5789 | 6181 | 6517 | 6853 | 7211 |
| 11010 | 1338* | 15286 | 15643 | 15970 | 16447 | 16813 | 17297 | 17770 | 18288 | 18825 | 19323 | 19931 | 25185 | 25912 | 4011 | 4290 | 4572 | 4940 | 5368 | 5792 | 6183 | 6518 | 6854 | 7216 |
| 11013 | 1343 | 15303 | 15646 | 15971 | 16450 | 16814 | 17300 | 17781 | 18294 | 18828 | 19325 | 19938 | 25205 | 25925 | 402 | 4291 | 4573 | 4942 | 5373 | 5794 | 6184 | 6525 | 6855 | 7219 |
| 11031 | 1346 | 15304* | 15653 | 15975 | 1646 | 16815 | 17301 | 17804 | 18299 | 18833 | 19327 | 19957 | 25230 | 26 | 4022 | 4293 | 4575 | 4944 | 5374 | 5795 | 6194 | 6526 | 6864 | 7234 |
| 11035 | 1347 | 15309 | 15664 | 15986 | 16463 | 16816 | 17303 | 17815 | 18303 | 18837 | 19330 | 1997 | 25231 | 260 | 4024 | 4294 | 4578 | 4951 | 5382 | 5797 | 6195 | 6527 | 6866 | 7240 |
| 11036 | 1348 | 15322 | 15669 | 15991 | 16465 | 16838 | 17311 | 17820 | 18307 | 18839 | 19332 | 19974 | 25232 | 26509 | 4027 | 4295 | 4580 | 4955 | 5388 | 5799 | 6199 | 6528 | 6879 | 7242 |
| 11043 | 1398* | 15326 | 15671 | 15997 | 16466 | 16841 | 17313 | 17826 | 18310 | 18848 | 19335 | 19981 | 25239 | 26527 | 4030 | 4296 | 4600 | 4958 | 5391 | 5800 | 6202 | 6533 | 6883 | 7244 |
| 11050 | 140 | 15329 | 15672 | 16000 | 16467 | 16848 | 17322 | 17829 | 18312 | 18849 | 19353 | 19982 | 25243 | 26528 | 4036 | 4297 | 4638* | 4961 | 5393 | 5802 | 6207 | 6538 | 6890 | 7246 |
| 11053 | 1403 | 15330 | 15680 | 16003 | 16468 | 16863 | 17325 | 17830 | 18319 | 1885 | 19357 | 19984 | 25244 | 26530 | 4038 | 4298* | 4670 | 4963 | 5402 | 5805 | 6212 | 6546 | 6901 | 7247 |
| 11055 | 141 | 15333 | 15681 | 16006 | 16471 | 16867 | 17331 | 17850 | 18324 | 18863* | 19363 | 19989 | 25263 | 26531 | 4039 | 4301 | 4671 | 4965 | 5404 | 5806 | 6223 | 6563 | 6906 | 7249 |
| 11056 | 1414* | 15340 | 15682 | 16009 | 16473 | 16869 | 17332 | 17853 | 18331 | 18864 | 19364 | 19996 | 25264 | 26532 | 4040 | 4303 | 4673 | 4967 | 5455 | 5810 | 6227 | 6565 | 6909 | 7264 |
| 11057 | 142 | 15346 | 15684 | 16012 | 16475 | 16870 | 17344 | 17857 | 18339 | 18871 | 19377 | 20014 | 25267 | 26534 | 4042 | 4304 | 4674 | 4968 | 5456 | 5814 | 6228 | 6566 | 6917 | 7281 |
| 11059 | 144 | 15363* | 15688 | 16018 | 16481 | 16874 | 17347 | 17863 | 18348 | 18878 | 19385 | 20020 | 25270 | 26543 | 4043 | 4308 | 4675 | 4969 | 5457 | 5816 | 6229 | 6571 | 6921 | 7285 |
| 11062 | 146 | 15364 | 15690 | 16034 | 16483 | 16876 | 17350 | 17864 | 18353 | 18884 | 19408 | 20026 | 25278 | 26548 | 4044 | 4311 | 4680 | 4973 | 5458 | 5819 | 6230 | 6575 | 6928 | 7286 |
| 11063 | 1474 | 15369 | 15694 | 16042 | 16487 | 16880 | 17362 | 17879 | 18371 | 18885 | 19417 | 20027 | 25280 | 26549 | 4045 | 4314* | 4683 | 4974 | 5462 | 5823 | 6237 | 6576 | 6930 | 7289* |
| 11064 | 1478 | 15372 | 15703 | 16043 | 16495* | 16883 | 17378 | 17902 | 18372 | 18889 | 19419 | 20028 | 25282 | 26551 | 4046 | 4316 | 4686 | 4978 | 5468 | 5832 | 6240 | 6579 | 6932 | 7290* |
| 11067 | 1479* | 15379 | 15706 | 16052 | 16508 | 16885 | 17384 | 17905 | 18377 | 18890 | 19421 | 20047 | 25292 | 26552 | 4048 | 4317 | 4698 | 4981 | 5471 | 5837 | 6252 | 6582 | 6936 | 7292 |
| 11070 | 1483 | 15383 | 15715 | 16059 | 16510 | 16890 | 17385 | 17907 | 18386 | 18895 | 19424 | 20049 | 25301 | 26558 | 4049 | 4318 | 4699 | 4983 | 5472* | 5838 | 6253 | 6585 | 6939 | 7296 |
| 11075 | 1495 | 15385 | 15719 | 16062 | 16516 | 16902 | 17392 | 17909 | 18403 | 18899 | 19426 | 20051* | 25305 | 26562 | 4055 | 4321 | 4703 | 4985 | 5474 | 5840 | 6255 | 6587 | 6940 | 7303 |
| 11082 | 1497 | 15389 | 15720 | 16067 | 16518 | 16910 | 17404 | 17916 | 18414 | 18928 | 19429 | 20075 | 25327 | 26564 | 4060 | 4323 | 4704 | 4990 | 5478 | 5846 | 6256 | 6590 | 6947 | 7310 |
| 11089 | 1500 | 15402 | 15727 | 16069 | 16523 | 16914 | 17410 | 17917 | 18417 | 18947 | 19443 | 20078 | 25328 | 26565 | 4062 | 4327 | 4709 | 4992 | 5483 | 5853 | 6267 | 66 | 6949 | 7312 |
| 11114 | 15002 | 15403 | 15732 | 16105 | 16525 | 16916 | 17411 | 17920 | 18419 | 18961 | 19444 | 20082 | 25329 | 26569 | 4065 | 4328 | 4711 | 4999 | 5484 | 5854 | 6271 | 6600 | 6950 | 7325 |
| 11177 | 15011 | 15404 | 15737 | 16112 | 16538 | 16918 | 17416 | 17922 | 18428 | 18978 | 19451 | 20085 | 25332 | 26571 | 4069 | 4329 | 4712 | 5003 | 5493 | 5857 | 6275 | 6601 | 6962 | 7358 |
| 11249 | 15012 | 15406 | 15739 | 16113 | 16540 | 16921 | 17417 | 17930 | 18433 | 18990 | 19457 | 20102 | 25341 | 26582 | 407* | 4330 | 4713 | 5009 | 5497 | 5862 | 6277 | 6604 | 6966 | 7377 |
| 11258 | 15019 | 15409 | 15741 | 16115 | 16541 | 1693 | 17427 | 17938 | 18477 | 18991 | 19459 | 20103* | 25343 | 26589 | 4070 | 4331 | 4715 | 5011 | 55 | 5863 | 6278 | 6606 | 6968 | 7421 |
| 1128 | 15020 | 15412 | 15751 | 16124 | 16568 | 16937 | 17428 | 17943 | 18480 | 18993 | 19463 | 20105 | 25345 | 26591 | 4071 | 4332 | 4718 | 5013 | 5504 | 5871 | 6279 | 6607 | 6972 | 7428 |
| 11303 | 15034 | 15420 | 15756 | 16140 | 16571 | 16944 | 17430 | 17946 | 18488 | 18994 | 19473 | 20108 | 25346 | 26592 | 4072 | 4333 | 4719 | 5014 | 5505 | 5872 | 6280 | 6610 | 6973 | 7434 |
| 11381 | 15036 | 15422 | 15757 | 16144 | 16588 | 16949 | 17437 | 17949 | 18494 | 18999 | 19477 | 20114 | 25354 | 26599 | 4076 | 4335 | 4722 | 5019 | 5509 | 5884 | 6287 | 6617 | 6984 | 754 |
| 11382 | 15048 | 15425 | 15758 | 16146 | 16591 | 16961 | 17445 | 17950 | 18495 | 19* | 19478 | 20119 | 25355 | 26600 | 4078 | 4336 | 4726 | 5029 | 5523 | 5889 | 6288* | 6618 | 6985 | 780 |
| 11383 | 15049 | 15426 | 15760 | 16147 | 16599 | 16964 | 17446 | 17953 | 18496 | 19004 | 19487 | 2012 | 25356 | 26601 | 408* | 4339 | 4727 | 5032 | 5524 | 5897 | 6289 | 6621 | 6987 | 79 |
| 11384 | 15050 | 15441 | 15761 | 16149* | 16600 | 16966 | 17454 | 17955 | 18505 | 19006 | 19490 | 20123 | 25358 | 26602 | 4082 | 4340 | 4729 | 5039* | 5534 | 5898 | 6291 | 6628 | 699 | 80 |
| 11385 | 15057 | 15446 | 15766 | 16150 | 16607 | 16982 | 17467 | 17959 | 18508 | 19008 | 19491 | 20126 | 25359 | 26603 | 4083 | 4341 | 4732 | 5040 | 5539 | 5918 | 6292 | 664 | 6990 | 810 |
| 11386 | 15060 | 1545 | 15769 | 16154 | 16608 | 17003 | 17471 | 17960 | 18523 | 19010 | 19495 | 2013* | 25364 | 26607 | 4085 | 4343 | 4737 | 5054 | 5541 | 5921 | 6294* | 6640 | 6995 | 88 |
| 11388 | 15061 | 15452 | 15770 | 16159 | 16611 | 17019 | 17472 | 17967 | 18527 | 19011 | 19496 | 2024 | 25383 | 26609 | 4086 | 4344 | 4739 | 5056 | 5550 | 5930 | 6296* | 6643 | 6999 | 916 |
| 11389 | 15064 | 15459 | 15779 | 1616 | 16613 | 17029 | 17474 | 17970 | 18528 | 19037 | 19499 | 2039 | 25386 | 26610 | 4092 | 4348 | 4742 | 5058 | 5563 | 5931 | 6297 | 6646 | 7004 | 932 |
| 11390 | 15082 | 15468 | 15782 | 16160 | 16614 | 17042 | 17476 | 17978 | 18530 | 19043 | 19510 | 2049 | 25387 | 26615 | 4094 | 4349 | 4748 | 5061 | 5568 | 5936 | 63 | 6647 | 7020 | 979 |
| 11391 | 15087 | 15475 | 15784 | 16162 | 16618 | 17043 | 17479 | 17992 | 18537 | 19044 | 19514 | 2059 | 2539 | 26616 | 4095 | 4353 | 4755 | 5062 | 5571 | 5942 | 6305 | 6648 | 7022 |  |
| 11392 | 15092 | 15481 | 15787 | 16163 | 16619 | 17048 | 17487 | 17997 | 18562 | 19046 | 19518 | 2060 | 25391 | 26617 | 41 | 4360 | 476* | 5067 | 5573 | 5945 | 6308 | 6658 | 7023 |  |
| 11393 | 15094 | 15482 | 15788 | 16165 | 16621 | 17062 | 17490 | 17998 | 18565 | 19048 | 19524* | 21014* | 25393 | 26618 | 4100 | 4363 | 4761 | 5081 | 5591 | 5955 | 6309 | 667 | 7024 |  |
| 11394 | 15097 | 15483* | 15789 | 16166 | 16623 | 17073 | 17491 | 17999 | 18569 | 19051 | 19532 | 21015* | 25399 | 26631 | 4101 | 4369 | 4772 | 5090 | 5595 | 5973 | 6319 | 6680 | 7025 |  |
| 11395 | 15100 | 15486 | 15795 | 16167 | 1663 | 17075 | 17496 | 18005 | 18572 | 19054 | 19544 | 2118 | 25401 | 26638 | 4102 | 4370 | 4774 | 5093 | 5600 | 5974 | 6329 | 6685 | 7032 |  |
| 11396 | 15102 | 15488 | 15798 | 16174 | 16630 | 17080 | 17497 | 18013 | 18574 | 19061 | 19554 | 2151 | 25403 | 26641 | 4109* | 4371 | 4782 | 5098* | 5602 | 5976 | 6332 | 6687 | 7049 |  |
| 11397 | 15110 | 15497 | 15800 | 16175 | 16638 | 1709 | 17499 | 18023 | 18578 | 19063 | 19559 | 218 | 25407 | 26642 | 4111 | 4374 | 4784 | 5102 | 5603 | 5983 | 6336 | 6688 | 7050 |  |
| 11398 | 15121 | 15498 | 15802 | 16177 | 1664* | 17109 | 17503 | 18025 | 18580 | 19069 | 19569 | 2196* | 25417 | 26644 | 4112* | 4376 | 4786 | 5103 | 5605 | 5984 | 6345 | 6689 | 7055 |  |
| 11399 | 15123 | 15501 | 15803 | 16182 | 16664 | 17112 | 17505 | 18026 | 18585 | 19074 | 19584 | 2198 | 25421 | 26651 | 4113 | 4377 | 4787* | 5105 | 5606 | 5987 | 6346 | 6693 | 7059 |  |
| 114 | 15127 | 15505 | 15806 | 16198 | 16665 | 17113 | 17507 | 18029 | 18588 | 19078 | 19589 | 2200 | 25441 | 26653 | 4115 | 4380 | 4788 | 5107 | 5615 | 5992 | 6351 | 6695 | 7066 |  |
| 1140* | 15129 | 15517 | 15808 | 162* | 16666 | 17126 | 17510 | 18032 | 18595 | 19082 | 19593 | 2204* | 25447 | 26654 | 4117 | 4381 | 4793 | 512 | 5618 | 5999 | 6352 | 6700 | 7069 |  |
| 11401 | 15132 | 15518 | 15809 | 16201 | 16668 | 17130 | 17546 | 18042 | 18598 | 19084 | 19597 | 2224 | 25453 | 26664 | 4121 | 4382 | 4794 | 5126 | 5623 | 6* | 6353 | 6702 | 7071 |  |
| 11402 | 15135 | 15519 | 15813 | 16206 | 16670 | 17131 | 17549 | 18045 | 18599 | 19088 | 19598 | 2281 | 2546 | 26672 | 4128 | 4384 | 4796 | 5142 | 5626 | 60* | 6361 | 6710 | 7072 |  |
| 11404 | 15138 | 15521 | 15817 | 16210 | 16673 | 17133 | 17550 | 18092 | 186 | 19104 | 19609 | 2283 | 25462 | 26694 | 4132 | 4385 | 480* | 5149 | 5628 | 6000 | 6363 | 6712 | 7073 |  |
| 11405 | 15141 | 15529 | 15822 | 16213 | 16683 | 17134 | 17552 | 18096 | 18601 | 19109 | 19611 | 2327 | 25483 | 26701 | 4134* | 4388 | 4817 | 5156 | 5629 | 6003 | 6364 | 6713 | 7080 |  |
| 11409 | 1515 | 15530 | 15830 | 16241* | 16685 | 17135 | 17555 | 18105 | 18604 | 19110 | 19638 | 2384 | 25485 | 26708 | 4136 | 4389 | 4818 | 5161 | 5630 | 6013 | 6368 | 6718 | 7081 |  |
| 11414 | 15150 | 15531 | 15831 | 16259 | 16689 | 17136 | 1756 | 1811* | 18615 | 19112 | 19641 | 2395 | 25501 | 26717 | 4138 | 4393 | 4819 | 5163 | 5650 | 6016 | 6371 | 6723 | 7082 |  |
| 11415 | 15162 | 15534 | 15832 | 16261 | 16690 | 17137 | 17569 | 18114 | 18627 | 19114 | 19653 | 2396 | 25512 | 26721 | 4142 | 440* | 4820 | 5182 | 5651 | 6021 | 6372 | 6724 | 7086 |  |
| 11420 | 15163 | 15537 | 15840 | 16262 | 16694 | 17141 | 17577 | 18122 | 18631 | 19135 | 19657 | 245 | 25533 | 275 | 4143 | 4403 | 4821 | 5190 | 5653 | 6025 | 6373 | 6732 | 7091 |  |
| 11427 | 1517 | 15539 | 15846 | 16266 | 16695 | 17146 | 17579 | 18124 | 18647 | 19138 | 19669 | 2474 | 25536 | 280 | 4145 | 4404 | 4827 | 5191 | 5658 | 6031 | 6375 | 6736 | 7094 |  |
| 11431 | 15171 | 15541 | 15848 | 16279 | 16696 | 17151 | 17589 | 18133 | 18664 | 19150 | 19672 | 248 | 25549 | 287 | 4149 | 4410 | 4832* | 5200 | 5661 | 6050 | 6393 | 6744 | 7103 |  |
| 11434 | 15172 | 15545 | 15866 | 16294 | 16698 | 17165 | 17592 | 18138 | 18667 | 19152 | 19704 | 250 | 25556 | 289* | 4150 | 4411* | 4834 | 5212 | 5663 | 6051 | 6404 | 6747 | 7105 |  |
| 11441 | 15173 | 15548 | 15871 | 16296 | 16703 | 17170 | 17597 | 18151 | 18671 | 19164 | 19708 | 25029 | 25557 | 2940* | 4158 | 4421 | 4836 | 5218 | 5665 | 6054 | 6405 | 6750 | 7107 |  |
| 11442 | 15178 | 15549 | 15874 | 16299 | 16710 | 17177 | 17599 | 18155 | 18676 | 19165 | 19715 | 25041 | 25572* | 2999 | 4159 | 4423 | 4839 | 5219 | 5668 | 6055 | 6406 | 6758 | 7115 |  |
| 11467 | 15179 | 15554 | 15875 | 16303 | 16716 | 17189 | 17602 | 18157 | 18680 | 19171 | 19723 | 25042 | 25592 | 30 | 4160 | 4436 | 4844 | 5226 | 5679 | 6057 | 6418 | 6771 | 7118 |  |

*Entries used in the calibration at acidic pH

| **Table S4. BMRB and relative PDB codes used in the benchmark of PROSECCO*_FOLDED_***. | | | | | |
| --- | --- | --- | --- | --- | --- |
| **BMRB ID** | **PDB ID** | **BMRB ID** | **PDB ID** | **BMRB ID** | **PDB ID** |
| 11609 | 2RVQ | 26066 | 2NDN | 34000 | 5L3L |
| 25664 | 2N41 | 26068 | 2NDP | 34009 | 5LAM |
| 25871 | 2N8Z | 26725 | 5IE8 | 34013 | 5LCI |
| 25939 | 2NAJ | 30003 | 5HP0 | 34018 | 5LFI |
| 25943 | 2NAQ | 30004 | 5HPD | 34022 | 5LG9 |
| 25944 | 2NAR | 30006 | 5HV8 | 34029 | 5LMY |
| 25947 | 2NAU | 30008 | 5I1R | 34043 | 5LW8 |
| 25948 | 2NAV | 30009 | 5I1X | 34050 | 5M1H |
| 25949 | 2NAW | 30010 | 5I22 | 34067 | 5MF9 |
| 25956 | 5FRH | 30020 | 5IAZ |  |  |
| 25957 | 2NB0 | 30021 | 5ID3 |  |  |
| 25958 | 2NB1 | 30023 | 5IEB |  |  |
| 25959 | 2NB2 | 30031 | 5IM8 |  |  |
| 25962 | 2NB5 | 30033 | 5IPO |  |  |
| 25963 | 2NB6 | 30034 | 5IRD |  |  |
| 25966 | 2NB8 | 30047 | 5J17 |  |  |
| 25967 | 2NB9 | 30070 | 5JN6 |  |  |
| 25968 | 5MMU | 30074 | 5JPW |  |  |
| 25969 | 2NBA | 30079 | 5JTK |  |  |
| 25976 | 2NBH | 30080 | 5JTL |  |  |
| 25985 | 2NBQ | 30102 | 5KES |  |  |
| 25994 | 2NBS | 30118 | 5KIZ |  |  |
| 26002 | 5FZV | 30126 | 5KNW |  |  |
| 26003 | 5FZW | 30127 | 5KP0 |  |  |
| 26004 | 5FZX | 30128 | 5KPE |  |  |
| 26010 | 2NC8 | 30129 | 5KPH |  |  |
| 26021 | 2NCG | 30134 | 5KRW |  |  |
| 26026 | 2NCJ | 30139 | 5KVP |  |  |
| 26031 | 2NCL | 30157 | 5T17 |  |  |
| 26041 | 2NCZ | 30161 | 5T3Y |  |  |
| 26045 | 2ND2 | 30177 | 5TBN |  |  |
| 26052 | 2ND9 | 30181 | 5TCZ |  |  |
| 26053 | 2NDA | 30200 | 5TTT |  |  |
| 26059 | 2NDF | 30205 | 5U3H |  |  |

| **Table S5. RMSDs (in ppm) of chemical shifts predicted using PROSECCO*_FOLDED_* for side chain atoms**. | | | | | | | | | | | | | | | | |
| --- | --- | --- | --- | --- | --- | --- | --- | --- | --- | --- | --- | --- | --- | --- | --- | --- |
| **ALA** | **HB** |  |  |  |  |  |  |  |  |  |  |  |  |  |  |  |
|  | 0.20 |  |  |  |  |  |  |  |  |  |  |  |  |  |  |  |
| **ARG** | **HB_(2/3)_** | **CG** | **HG_(2/3)_** | **CD** | **HD_(2/3)_** | **CZ** | **NE** | **HE** | **NH_(1/2)_** | **HH_(11/12/21/22)_** |  |  |  |  |  |  |
|  | 0.21 | 1.10 | 0.22 | 0.98 | 0.18 | -- | -- | 0.52 | -- | -- |  |  |  |  |  |  |
| **ASP** | **HB_(2/3)_** | **CG** | **HD2** |  |  |  |  |  |  |  |  |  |  |  |  |  |
|  | 0.21 | -- | -- |  |  |  |  |  |  |  |  |  |  |  |  |  |
| **ASN** | **HB_(2/3)_** | **CG** | **ND2** | **HD2_(1/2)_** |  |  |  |  |  |  |  |  |  |  |  |  |
|  | 0.26 | 1.00 | 2.11 | 0.43 |  |  |  |  |  |  |  |  |  |  |  |  |
| **CYS** | **HB_(2/3)_** | **HG** |  |  |  |  |  |  |  |  |  |  |  |  |  |  |
|  | 0.31 | 0.56 |  |  |  |  |  |  |  |  |  |  |  |  |  |  |
| **GLU** | **HB_(2/3)_** | **CG** | **HG_(2/3)_** | **CD** | **HE2** |  |  |  |  |  |  |  |  |  |  |  |
|  | 0.17 | 0.96 | 0.17 | -- | -- |  |  |  |  |  |  |  |  |  |  |  |
| **GLN** | **HB_(2/3)_** | **CG** | **HG_(2/3)_** | **CD** | **NE2** | **HE2_(1/2)_** |  |  |  |  |  |  |  |  |  |  |
|  | 0.20 | 0.95 | 0.25 | -- | 1.53 | 0.38 |  |  |  |  |  |  |  |  |  |  |
| **HIS** | **HB_(2/3)_** | **ND1** | **HD1** | **NE2** | **HE2** | **CG** | **CD2** | **HD2** | **CE1** | **HE1** |  |  |  |  |  |  |
|  | 0.30 | -- | -- | -- | -- | -- | -- | 0.40 | 1.52 | 0.43 |  |  |  |  |  |  |
| **ILE** | **HB_(2/3)_** | **CG1** | **HG1_(2/3)_** | **CD1** | **HD1** | **CG2** | **HG2** |  |  |  |  |  |  |  |  |  |
|  | 0.25 | 1.31 | 0.42 | 1.62 | 0.23 | 1.11 | 0.23 |  |  |  |  |  |  |  |  |  |
| **LEU** | **HB_(2/3)_** | **CG** | **HG** | **CD1** | **HD1** | **CD2** | **HD2** |  |  |  |  |  |  |  |  |  |
|  | 0.32 | 1.01 | 0.35 | 1.56 | 0.35 | 1.63 | 0.24 |  |  |  |  |  |  |  |  |  |
| **LYS** | **HB_(2/3)_** | **CG** | **HG_(2/3)_** | **CD** | **HD_(2/3)_** | **NZ** | **HZ** | **CE** | **HE_(2/3)_** |  |  |  |  |  |  |  |
|  | 0.21 | 1.14 | 0.23 | 0.94 | 0.20 | -- | -- | 0.61 | 0.16 |  |  |  |  |  |  |  |
| **MET** | **HB_(2/3)_** | **CG** | **HG_(2/3)_** | **CE** | **HE** |  |  |  |  |  |  |  |  |  |  |  |
|  | 0.35 | 1.03 | 0.35 | 1.66 | 0.26 |  |  |  |  |  |  |  |  |  |  |  |
| **PHE** | **HB_(2/3)_** | **CG** | **CD_(1/2)_** | **HD_(1/2)_** | **CE_(1/2)_** | **HE_(1/2)_** | **CZ** | **HZ** |  |  |  |  |  |  |  |  |
|  | 0.32 | -- | 1.15 | 0.27 | 1.15 | 0.32 | 1.48 | 0.36 |  |  |  |  |  |  |  |  |
| **PRO** | **CG** | **HG_(2/3)_** | **CD** | **HD_(2/3)_** |  |  |  |  |  |  |  |  |  |  |  |  |
|  | 0.88 | 0.23 | 0.79 | 0.31 |  |  |  |  |  |  |  |  |  |  |  |  |
| **SER** | **HB_(2/3)_** | **HG** |  |  |  |  |  |  |  |  |  |  |  |  |  |  |
|  | 0.21 | -- |  |  |  |  |  |  |  |  |  |  |  |  |  |  |
| **THR** | **HB** | **HG1** | **CG2** | **HG2** |  |  |  |  |  |  |  |  |  |  |  |  |
|  | 0.27 | 1.46 | 1.02 | 0.20 |  |  |  |  |  |  |  |  |  |  |  |  |
| **TRP** | **HB_(2/3)_** | **CG** | **CD1** | **HD1** | **NE1** | **HE1** | **CE3** | **HE3** | **CZ2** | **HZ2** | **CD2** | **CE2** | **CZ3** | **HZ3** | **CH2** | **HH2** |
|  | 0.24 | -- | 1.55 | 0.33 | 2.14 | 0.49 | 1.4 | 0.34 | 1.27 | 0.29 | -- | -- | 2.33 | 0.30 | 1.55 | 0.31 |
| **TYR** | **HB_(2/3)_** | **CG** | **CD_(1/2)_** | **HD_(1/2)_** | **CE_(1/2)_** | **HE_(1/2)_** | **CZ** | **HH** |  |  |  |  |  |  |  |  |
|  | 0.31 | -- | 1.62 | 0.29 | 1.28 | 0.2 | -- | -- |  |  |  |  |  |  |  |  |
| **VAL** | **HB** | **CG1** | **HG1** | **CG2** | **HG2** |  |  |  |  |  |  |  |  |  |  |  |
|  | 0.26 | 1.26 | 0.21 | 1.41 | 0.27 |  |  |  |  |  |  |  |  |  |  |  |

-- Atoms with too few observations in the BMRB to yield a prediction.

Cys residues are treated as reduced by default, His as unprotonated and Pro as cis

**SI-References**

Jones, D.T. (1999). Protein secondary structure prediction based on position-specific scoring matrices. J. Mol. Biol. *292*, 195–202.

Kohlhoff, K.J., Robustelli, P., Cavalli, A., Salvatella, X., and Vendruscolo, M. (2009). Fast and accurate predictions of protein NMR chemical shifts from interatomic distances. J. Am. Chem. Soc. *131*, 13894–13895.

Sahakyan, A.B., Vranken, W.F., Cavalli, A., and Vendruscolo, M. (2011). Structure-based prediction of methyl chemical shifts in proteins. J. Biomol. NMR *50*, 331–346.

Shen, Y., and Bax, A. (2010). SPARTA+: A modest improvement in empirical NMR chemical shift prediction by means of an artificial neural network. J. Biomol. NMR *48*, 13–22.

Shen, Y., Vernon, R., Baker, D., and Bax, A. (2009). De novo protein structure generation from incomplete chemical shift assignments. J. Biomol. NMR *43*, 63–78.
